# Association of the glucose-to-lymphocyte ratio with all-cause and cardiovascular disease mortality in U.S. adults: A population-based cohort study

**DOI:** 10.1016/j.clinsp.2026.101117

**Published:** 2026-09-09

**Authors:** Yongtong He, Qiyin Cai, Songbai Wang

**Affiliations:** a Department of Neurosurgery, Affiliated Jiangmen Traditional Chinese Medicine (TCM) Hospital of Ji'nan University, Jiangmen, China; b Department of Pediatric intensive care units, Jiangmen Maternity and Child health care hospital, Jiangmen, China; c Department of Hospital Administration, Jiangmen Maternity and Child health care hospital, Jiangmen, China

**Keywords:** Glucose-to-lymphocyte ratio, Mortality, Cardiovascular disease, NHANES, Metabolic-immune marker, Population-based cohort

## Abstract

**Background:**

The Glucose-to-Lymphocyte Ratio (GLR) reflects metabolic-immune status, but its association with mortality in the general population remains unclear.

**Methods:**

We analyzed 22,120 adults from NHANES (1999–2016) with complete data on glucose, lymphocytes and mortality. Associations between log₂-transformed GLR and all-cause or Cardiovascular Disease (CVD) mortality were evaluated using multivariable Cox models. Nonlinear dose–response relationships were assessed with restricted cubic splines, and survival differences visualized with Kaplan-Meier curves. Model performance was compared using ROC curves and DCA for inflammatory markers, while NRI and IDI were calculated based on the Framingham risk model.

**Results:**

Higher GLR was associated with older age, comorbidities, and increased mortality over a median follow-up of 122-months. Fully adjusted models showed associations with all-cause (HR = 1.33, 95% CI 1.25–1.42) and CVD mortality (HR = 1.51, 95% CI 1.36–1.66). Dose–response analyses indicated J-shaped curves with exploratory, cohort-specific inflection points at GLR ≈46 (all-cause) and ≈49 (CVD). GLR showed comparable or slightly better discrimination (AUC 67.5%‒67.7%) than individual inflammatory markers. When added to the Framingham model, GLR provided modest improvements in reclassification indices (NRI and IDI). E-values, subgroup and sensitivity analyses, and competing risks models showed the model was robust.

**Conclusion:**

In this study, elevated GLR was associated with higher all-cause and CVD mortality in a J-shaped manner. While these findings suggest GLR may be a composite metabolic-immune marker worthy of further investigation, its clinical utility remains uncertain and requires external validation.

## Highlights

• Elevated GLR is independently associated with higher all-cause and CVD mortality in a J-shaped manner.
• GLR showed comparable or modestly better discrimination than individual inflammatory biomarkers.
• The association persisted across sensitivity, competing-risk, and subgroup analyses after multiple-testing correction.
• GLR improved risk reclassification when added to a basic Framingham risk model.

## Introduction

Current scientific investigations prioritize identifying biomarkers that integrate mechanistic insights into systemic stress and immune dysregulation and may assist in risk stratification of mortality. Circulating indicators such as lipopolysaccharide-binding protein,^1^ superoxide dismutase,^2^ and pro-brain natriuretic peptide^3^ have gained prominence for their prognostic capabilities regarding all-cause mortality. However, healthcare systems increasingly need simple, cost-effective prognostic tools that fit routine clinical workflows.

Routine lab measures of glucose and lymphocyte count offer key insights into systemic health. Hyperglycemia is linked to oxidative stress, insulin issues, and inflammation ‒ factors driving multiorgan dysfunction.^4^ Lymphocyte levels reflect immune status; lymphopenia is consistently tied to weaker host defense.^5^ Clinical evidence consistently associates lymphopenia with diminished host defense capacity and elevated susceptibility to infections/poor prognoses.^6^ a pattern markedly observed in critical care settings through correlations between depressed lymphocyte levels and increased acute mortality risks.^7^

Although arising from distinct biological pathways, glucose and lymphocyte count can jointly reflect oxidative stress, inflammation, and immune regulation.^8^ The Glucose-to-Lymphocyte Ratio (GLR) is conceptually analogous to established heterogeneous-unit ratios such as Neutrophil-to-Lymphocyte (NLR) and Platelet-to-Lymphocyte (PLR) ratios, capturing reciprocal metabolic-immune dynamics rather than functioning as a standalone inflammatory marker. Specifically, GLR integrates two coupled coordinated consequences of systemic stress: cortisol- and catecholamine-driven hyperglycemia, and β-adrenergic/CXCR4-mediated lymphocyte redistribution.^9^ Hyperglycemia activates NF-κB, promoting IL-6 release and endothelial dysfunction.^10^ Together, these tightly coupled processes form a coordinated signature of metabolic strain and immune suppression.^11^ Division is chosen because it reflects the relative dominance of metabolic over immune arm: when glucose rises faster than lymphocyte fall, the ratio amplifies the signal of systemic allostatic load. This makes the index more physiologically interpretable than subtraction or multiplication, and more robust to inter-individual variation in baseline levels. GLR condenses this imbalance into a dimensionless composite index of allostatic load,^12^ capturing the relative magnitude of stress-induced hyperglycemia and immune cell redistribution.

Importantly, the mathematical form of GLR is biologically meaningful. A ratio amplifies the opposing directions of two stress-responsive biomarkers ‒ glucose rising while lymphocytes fall ‒ thereby quantifying the relative dominance of metabolic over immune dysregulation.^13^ Moreover, because it was pre-specified based on mechanistic rationale, concerns regarding “ratio fishing” are substantially reduced.^14^ Integrating glucose and lymphocyte parameters may therefore provide a pragmatic summary of prognostic accuracy for mortality through their complementary roles in homeostasis and pathophysiology.^15^ Because GLR is calculated from ubiquitous laboratory tests, it offers greater clinical practicality than isolated biomarker interpretation. Emerging evidence suggests prognostic utility of GLR in renal impairment,^16^ sepsis,^17^ and cancer.^18^ However, its association with all-cause and cardiovascular mortality at the population level remains insufficiently defined, and the biological plausibility of this specific ratio warrants clearer articulation.

To address this gap, we hypothesized that elevated GLR would be associated with higher mortality risk in the general population and evaluated this using nationally representative data from the National Health and Nutrition Examination Survey (NHANES).

## Methods

### Study population

NHANES offers a thorough cross–sectional evaluation of the nutritional status and general health of the US civilian population that is not institutionalized. At Mobile Examination Centers (MEC), participants had a standardized physical examination conducted by qualified specialists, and the official NHANES website (https://www.cdc.gov/nchs/nhanes/?CDC_AAref_Val) has comprehensive details on the survey's methodology and data gathering processes. Data gathered from 1999 to 2016 were examined for this cohort study, and mortality results were verified by comparing them to the National Death Index (https://www.cdc.gov/nchs/ndi/index.html).

**Fig. 1:**
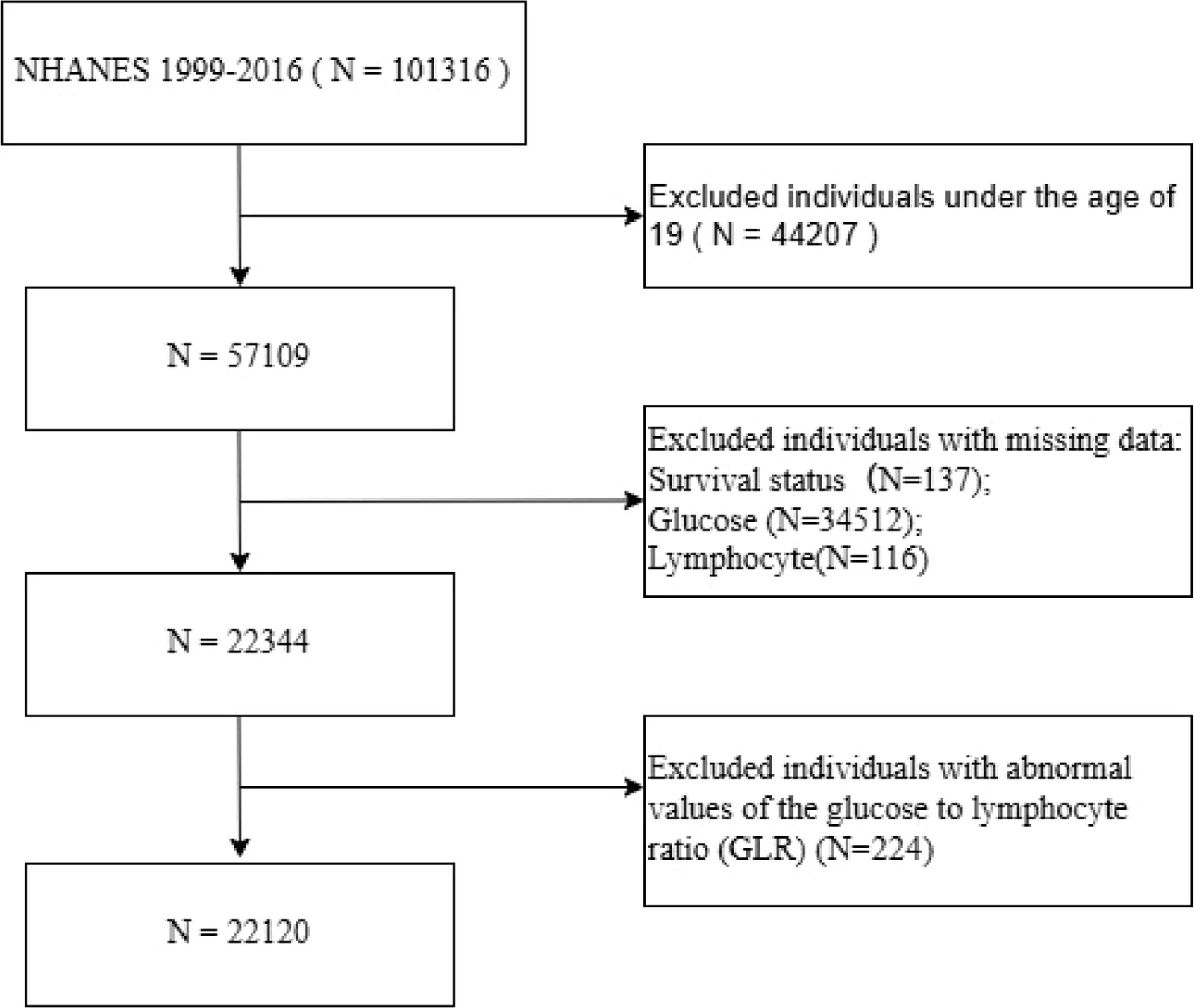
Flowchart of participant selection.

Our analysis cohort initially comprised 101,316 NHANES participants (1999–2016). We excluded individuals aged < 19-years (n = 44,207) and implemented rigorous data quality controls: 1) Exclusion of cases with missing survival status (n = 137), 2) Removal of participants lacking glucose (n = 34,512) or lymphocyte count (n = 116), 3) Truncation of extreme GLR values beyond the 0.5th‒99.5th percentiles^19^ (n = 224) were removed from the study. Ultimately, the final sample comprised 22,120 participants (Fig. 1).

Informed consent was obtained in writing from all participants, and the research was approved by the NCHS Research Ethics Review Board (http://www.cdc.gov/nchs/nhanes/about/erb.html; The ethics committee study protocol numbers are #98–12 (1999–2004), #2005–06 (2005–2010), and #2011–17 (2011–2016). The study adhered to the guidelines established by the Strengthening the Reporting of Observational Studies in Epidemiology (STROBE).

### Measurement of GLR

GLR, obtained directly from laboratory records, is calculated by dividing the glucose level (mg/dL) by the lymphocyte count (cells/µL)^20^ from the antecubital vein of each participant by a trained phlebotomist following the MEC protocol. Detailed information regarding laboratory testing protocols can be found in the NHANES Laboratory/Medical Technician Procedures Manual. Exposure assessment and cohort entry occurred simultaneously at the MEC visit, with follow-up beginning immediately thereafter, ensuring no temporal gap between exposure definition and follow-up and thereby eliminating immortal time bias.

### Outcomes

All-cause mortality was the study's main outcome, while Cardiovascular Disease (CVD) mortality was its secondary. Unless they left the study or met the endpoint, NHANES participants were tracked until December 31, 2019. Mortality status was confirmed using data from the National Death Index. According to the International Classification of Diseases, 10th Revision, Clinical Modification System codes (ICD–10 codes I00 to I09, I11, I13, I20 to I51, and I60 to I69), CVD mortality was defined as death from cardiovascular or cerebrovascular disease.^21^

### Covariates

Covariates were selected a priori based on reported associations with GLR and mortality. Demographic, lifestyle, and clinical data were obtained via interviews and MEC examinations.^22^ Age, sex, race and ethnicity, educational status, Body Mass Index (BMI), and Poverty Income Ratio (PIR) were included. Participants were categorized by age (45, 45–64, ≥ 65-years), sex (male/female), race/ethnicity (Mexican American, Hispanic, non-Hispanic White, non-Hispanic Black, other), education status (< high school, high school, > high school), poverty-income ratio (low < 1.3, medium 1.3–3.4, high ≥ 3.5), and BMI (underweight < 18.5, normal 18.5–24.9, overweight 25–29.9, obese ≥ 30 kg/m^2^). Lifestyle factors included smoking (≥ 100 lifetime cigarettes), alcohol use (≥ 12 drinks/year),^23^ and physical activity (low, moderate, or vigorous-intensity).^24^ Self-reported physician-diagnosed hypertension, stroke, or Coronary Heart Disease (CHD), cancer, and diabetes were included.^25^ In addition, renal function (Blood Urea Nitrogen [BUN], creatinine), blood lipids (Total Cholesterol [TC], High-Density Lipoprotein-Cholesterol [HDL-C]), antidiabetic medication use, antihypertensive use, and statin use were also incorporated as confounders.

### Statistical analysis

Firstly, continuous variables were subjected to a normality assessment. Due to their skewed distribution, GLR values were analyzed both as continuous (log_2_-transformed) data and categorized into tertiles (T1 < 45.66, T2 = 45.66–61.17, T3 ≥ 61.18) in the overall study cohort. Categorical variables were described using counts and corresponding percentages. Groups were assessed using the *t*-test, one-way ANOVA, the Kruskal-Wallis H-test, or the Chi-Square test as appropriate. For variables with under 20% missingness, multivariate imputation by chained equations (MICE, 5-imputations, including all covariates in the imputation model) was performed to reduce bias^26^ (Table S1).

Secondly, log_2_-transformed GLR (continuous) was analyzed as using Cox proportional hazard regression to estimate Hazard Ratios (HRs) and 95% Confidence Intervals (95% CIs) for all-cause and CVD mortality. Covariates for the multivariable models were selected based on prior studies and as well as components of the Framingham Risk Score.^27^ Four models were fitted: Model 1 was unadjusted (crude model); Model 2 adjusted for demographic factors, including age (continuous), sex, education status, race and ethnicity, and PIR; Model 3 further included alcohol consumption, smoking status, BMI, hypertension, stroke, CHD, and cancer; and Model 4 (fully adjusted) additionally incorporated renal function (creatinine, BUN), blood lipids (TC, HDL-C), antidiabetic medication use, antihypertensive use, statin use, and physical activity.^28^ Diabetes was not included as GLR is derived from blood glucose, avoiding overadjustment. Systolic blood pressure was omitted, as it likely mediates the metabolic–cardiovascular pathway rather than confounding it (Fig. S1), in contrast, hypertension diagnosis and antihypertensive use, as upstream factors, were retained. This choice was supported by supplementary mediation analyses. Furthermore, trend tests were performed. The proportional hazards assumption was assessed using scaled Schoenfeld residuals.

After multivariable model fitting, we assessed model performance through discrimination metrics, calibration analysis, Decision Curve Analysis (DCA), and internal validation via 100 repetitions of 5-fold cross-validation. To account for competing risks, Fine-Gray sub distribution hazard models were fitted non-CVD deaths treated as competing events.

Thirdly, nonlinear associations were assessed using restricted cubic splines (4 knots at 5th, 35th, 65th, and 95th percentiles) and two-piecewise linear regression to explore threshold effects. A two-piecewise linear regression model was built, based on smoothed curves, to investigate potential threshold effects. Kaplan-Meier survival curves were generated to visualize differences in survival probabilities.

Fourthly, subgroup analyses were conducted by age categories, sex, race and ethnicity, alcohol consumption, BMI (25 kg/m^2^ vs. ≥ 25 kg/m^2^), and the presence of hypertension, diabetes and CHD, with interactions tested using likelihood ratio tests. p-values for multiple testing were adjusted using Bonferroni correction, with adjusted p*-*value < 0.05 considered significant. These subgroup analyses were considered exploratory.

In sensitivity analyses, we repeated Cox regression to test the robustness of our findings using datasets including outlier cases and pre-imputation data. Additional sensitivity analyses incorporating diabetes as a covariate, employed different GLR categorization approaches (quartiles and quintiles), and applying various imputation methods (simple substitution [median (continuous)/mode (categorical)], dummy variable adjustment, and decision-tree-based imputation). We also performed analyses excluding extreme values (±3SD) and participants with CHD. Two-sided p-value < 0.05 was considered statistically significant.

Finally, GLR was examined alongside established inflammatory markers, including glucose, lymphocyte count, NLR, PLR, C-Reactive Protein (CRP). Area Under the Curve (AUC) values with 95% CIs were calculated using the DeLong method. DCA illustrated the observed ranges of marker values in relation to mortality. analyses were restricted to participants with complete biomarker data between 2003 and 2011, when platelet counts and CRP were routinely measured. GLR was also incorporated descriptively into Framingham risk model, with Net Reclassification Improvement (NRI) and Integrated Discrimination Improvement (IDI) reported for completeness.

E-value analysis was performed to quantify the minimum strength of an unmeasured confounder required to explain the observed associations.

Analysis was performed using R 4.2.2 (http://www.Rproject.org; The R Foundation, Vienna, Austria) and the Free Statistics software (version 2.0; Beijing Free Clinical Medical Technology Co., Ltd, Beijing, China).

## Results

### Characteristics of the study population

This study includes a total of 22,120 participants from the NHANES dataset. Table 1 presents the baseline characteristics of these eligible participants. The cohort comprised 44.9% individuals aged under 45-years old with a balanced sex distribution (51.8% female and 48.2% male), and diverse racial composition (45.1% White and 20.0% Black). Hypertension, stroke, CHD, cancer and diabetes had overall prevalence rates of 27.6%, 3.6%, 3.9%, 8.7%, and 11.0%, respectively. Participants' average age was 48.5 ± 18.8 years, individuals with elevated GLR were older and more likely to have chronic diseases, particularly diabetes (T3: 22.3% vs. T1: 4.2%) and hypertension (T3: 37.6% vs. T1: 20.1%). Death rates from all-cause and CVD were 16.4% and 5.2%, respectively, across a median follow-up of 122-months. Mortality risks showed strong GLR-dependent gradients: all-cause (T3: 26.3% vs. T1: 9.3%) and CVD mortality (T3: 9.2% vs. T1: 2.5%) were approximately 3-fold higher in the highest versus lowest GLR tertile.

**Table 1:** Baseline clinical characteristics of the study population.

Characteristic	Overall (n = 22,120)	GLR			p-value
		T1, <45.66 (n = 7373)	T2, 45.66‒61.17 (n = 7356)	T3, ≥61.18 (n = 7391)	
Age (years), Mean ± SD	48.5 ± 18.8	41.3 ± 17.3	47.3 ± 18.2	56.9 ± 17.4	<0.001
Age category (years), n (%)					<0.001
< 45	9922 (44.9)	4526 (61.4)	3523 (47.9)	1873 (25.3)	
45‒64	6941 (31.4)	1957 (26.5)	2293 (31.2)	2691 (36.4)	
≥ 65	5257 (23.8)	890 (12.1)	1540 (20.9)	2827 (38.2)	
Sex, n (%)					<0.001
Male	10,665 (48.2)	3147 (42.7)	3450 (46.9)	4068 (55)	
Female	11,455 (51.8)	4226 (57.3)	3906 (53.1)	3323 (45)	
Race and ethnicity, n (%)					<0.001
Mexican American	4112 (18.6)	1449 (19.7)	1442 (19.6)	1221 (16.5)	
Hispanic	1834 (8.3)	662 (9)	594 (8.1)	578 (7.8)	
White	9981 (45.1)	2900 (39.3)	3350 (45.5)	3731 (50.5)	
Black	4422 (20.0)	1725 (23.4)	1353 (18.4)	1344 (18.2)	
Other	1771 (8.0)	637 (8.6)	617 (8.4)	517 (7)	
Educational status, n (%)					<0.001
Below high school	2773 (12.5)	819 (11.1)	918 (12.5)	1036 (14)	
High school	8501 (38.4)	3065 (41.6)	2662 (36.2)	2774 (37.5)	
Above high school	10,846 (49.0)	3489 (47.3)	3776 (51.3)	3581 (48.5)	
PIR, n (%)					<0.001
Low income	7072 (32.0)	2680 (36.3)	2271 (30.9)	2121 (28.7)	
Medium income	8415 (38.0)	2780 (37.7)	2781 (37.8)	2854 (38.6)	
High income	6633 (30.0)	1913 (25.9)	2304 (31.3)	2416 (32.7)	
BMI (kg/m^2^), n (%)					<0.001
Underweight (18.5)	392 (1.8)	168 (2.3)	107 (1.5)	117 (1.6)	
Normal (18.5‒24.9)	6581 (29.8)	2245 (30.4)	2308 (31.4)	2028 (27.4)	
Overweight (25‒29.9)	7462 (33.7)	2343 (31.8)	2533 (34.4)	2586 (35)	
Obese (≥ 30)	7685 (34.7)	2617 (35.5)	2408 (32.7)	2660 (36)	
Alcohol consumption, n (%)	15,434 (69.8)	5245 (71.1)	5164 (70.2)	5025 (68)	<0.001
Smoke status, n (%)					<0.001
Never	11,868 (53.7)	3817 (51.8)	4103 (55.8)	3948 (53.4)	
Former	5600 (25.3)	1361 (18.5)	1839 (25)	2400 (32.5)	
Current	4652 (21.0)	2195 (29.8)	1414 (19.2)	1043 (14.1)	
Hypertension, n (%)	6100 (27.6)	1479 (20.1)	1839 (25)	2782 (37.6)	<0.001
Stroke, n (%)	787 (3.6)	172 (2.3)	216 (2.9)	399 (5.4)	<0.001
CHD, n (%)	869 (3.9)	125 (1.7)	243 (3.3)	501 (6.8)	<0.001
Cancer, n (%)	1916 (8.7)	387 (5.2)	550 (7.5)	979 (13.2)	<0.001
Diabetes, n (%)	2428 (11.0)	313 (4.2)	468 (6.4)	1647 (22.3)	<0.001
Creatinine (mg/dL)	0.8 (0.7, 1.0)	0.8 (0.7, 0.9)	0.8 (0.7, 1.0)	0.9 (0.7, 1.0)	<0.001
BUN (mg/dL)	13.4 ± 6.0	12.1 ± 5.0	13.0 ± 5.2	15.0 ± 7.2	<0.001
TC (mg/dL)	195.0 ± 41.9	195.3 ± 42.6	196.0 ± 40.8	193.8 ± 42.4	0.006
HDL-C (mg/dL)	53.7 ± 16.1	52.8 ± 15.7	54.3 ± 15.9	54.0 ± 16.7	<0.001
Antidiabetic medication use, n (%)	1760 (8.0)	236 (3.2)	335 (4.6)	1189 (16.1)	<0.001
Antihypertensive use, n (%)	4863 (22.0)	1205 (16.3)	1446 (19.7)	2212 (29.9)	<0.001
Statin use, n (%)	2819 (12.7)	526 (7.1)	799 (10.9)	1494 (20.2)	<0.001
Physical activity, n (%)					<0.001
Low-intensity	11,389 (53.5)	3610 (52.1)	3763 (53.2)	4016 (55.1)	
Moderate-intensity	2424 (11.4)	769 (11.1)	801 (11.3)	854 (11.7)	
Vigorous-intensity	7479 (35.1)	2551 (36.8)	2513 (35.5)	2415 (33.2)	
All-cause mortality, n (%)	3634 (16.4)	686 (9.3)	1002 (13.6)	1946 (26.3)	<0.001
CVD mortality, n (%)	1161 (5.2)	188 (2.5)	294 (4)	679 (9.2)	<0.001
Follow-up time (months), Median (IQR)	122.0 (75.0, 177.0)	129.0 (80.0, 184.0)	124.0 (79.0, 181.0)	111.0 (68.0, 164.0)	<0.001

BMI, Body Mass Index; PIR, Poverty-Income Ratio; GLR, Glucose to Lymphocyte Ratio; CHD, Coronary Heart Disease; BUN, Blood Urea Nitrogen; TC, Total Cholesterol; HDL-C, High-Density Lipoprotein Cholesterol; CVD, Cardiovascular Disease.

### Associations between GLR and mortality

Table 2 shows the associations between GLR and all-cause and CVD mortality across four multivariable models. In the unadjusted Model 1, each unit increase in log_2_-transformed GLR was associated with significantly higher risks (all-cause: HR = 2.74, 95% CI 2.59–2.89; CVD: HR = 3.33, 95% CI 3.03–3.66; both p < 0.001). Adjustment for demographics in Model 2 attenuated the associations (all-cause: HR = 1.35, 95% CI 1.27–1.43; CVD: HR = 1.60, 95% CI 1.44–1.78), and further adjustment for clinical factors in Model 3 yielded similar results (all-cause: HR = 1.38, 95% CI 1.30–1.47; CVD: HR = 1.59, 95% CI 1.43–1.77). In the fully adjusted Model 4, including renal function, blood lipids, medications, and physical activity, associations remained significant (all-cause: HR = 1.33, 95% CI 1.25–1.42; T3 vs. T1: HR = 1.20, 95% CI 1.10–1.32; CVD: HR = 1.51, 95% CI 1.36–1.66; T3 vs. T1: HR = 1.33, 95% CI 1.13–1.58). Across all models, participants in the highest GLR tertile consistently had higher risks than those in the lowest tertile (p for trend < 0.001). The proportional hazards assumption was evaluated using scaled Schoenfeld residuals, and no significant violations were detected for either all-cause or CVD mortality (Table S2), supporting the validity of the Cox model estimates.

**Table 2:** Multivariate logistic regression analysis of glucose to lymphocyte ratio and All-cause and CVD mortality.

Variable	N° of events	Model 1	p*-*value	Model 2	p*-*value	Model 3	p*-*value	Model 4	p*-*value
		HR (95% CI)		HR (95% CI)		HR (95% CI)		HR (95% CI)	
**All-cause mortality**									
GLR (log_2_) continuous	22,120	2.74 (2.59–2.89)	<0.001	1.35 (1.27–1.43)	<0.001	1.38 (1.30–1.47)	<0.001	1.33 (1.25–1.42)	<0.001
GLR (log2) tertile									
T1	7373	1 (Ref)		1 (Ref)		Ref		Ref	
T2	7356	1.50 (1.37–1.66)	<0.001	0.94 (0.85–1.04)	0.24	0.99 (0.90–1.10)	0.887	0.98 (0.89–1.08)	0.706
T3	7391	3.27 (3.00–3.57)	<0.001	1.19 (1.09–1.30)	<0.001	1.25 (1.14–1.37)	<0.001	1.20 (1.10–1.32)	<0.001
p*-*value for trend test			<0.001		<0.001		<0.001		<0.001
**CVD mortality**									
GLR (log_2_) continuous	22,120	3.33 (3.03–3.66)	<0.001	1.60 (1.44–1.78)	<0.001	1.59 (1.43–1.77)	<0.001	1.51 (1.36∼1.66)	<0.001
GLR (log2) tertile									
T1	7373	1 (Ref)		1 (Ref)		1 (Ref)		Ref	
T2	7356	1.61 (1.34–1.93)	<0.001	0.96 (0.8–1.16)	0.698	0.98 (0.81–1.18)	0.826	0.97 (0.81–1.18)	0.746
T3	7391	4.16 (3.54–4.89)	<0.001	1.39 (1.18–1.64)	<0.001	1.39 (1.17–1.64)	<0.001	1.33 (1.13–1.58)	0.001
p-value for trend test			<0.001		<0.001		<0.001		<0.001

Model 1: No adjusted covariates. Model 2: Adjusted for age, sex, race and ethnicity, educational status, PIR. Model 3: Adjusted for Model 2 plus BMI, alcohol consumption, smoke status, hypertension, stroke, coronary heart disease, cancer. Model 4: Adjusted for Model 3 plus creatinine, BUN, TC, HDL-C, antidiabetic medication use, antihypertensive use, statin use and physical activity.

GLR, Glucose to Lymphocyte Ratio; CVD, Cardiovascular Disease; BMI, Body Mass Index; PIR, Poverty-Income Ratio; CHD, Coronary Heart Disease; BUN, Blood Urea Nitrogen; TC, Total Cholesterol; HDL-C, High-Density Lipoprotein Cholesterol; HR, Hazard Ratios; CI, Confidence Interval.

Sensitivity analysis using the Fine-Gray sub distribution hazard model confirmed a significant positive association between GLR and cardiovascular mortality after full adjustment (Model 4). These findings were consistent with the primary Cox regression results, indicating that competing risks did not materially affect the observed associations (Table S3). Model diagnostics ‒ including discrimination, calibration, DCA, and repeated 5-fold cross-validation ‒ demonstrated stable model performance and supported the robustness of the findings. (Table S4‒S5, Fig. S2‒S3).

**Fig. 2:**
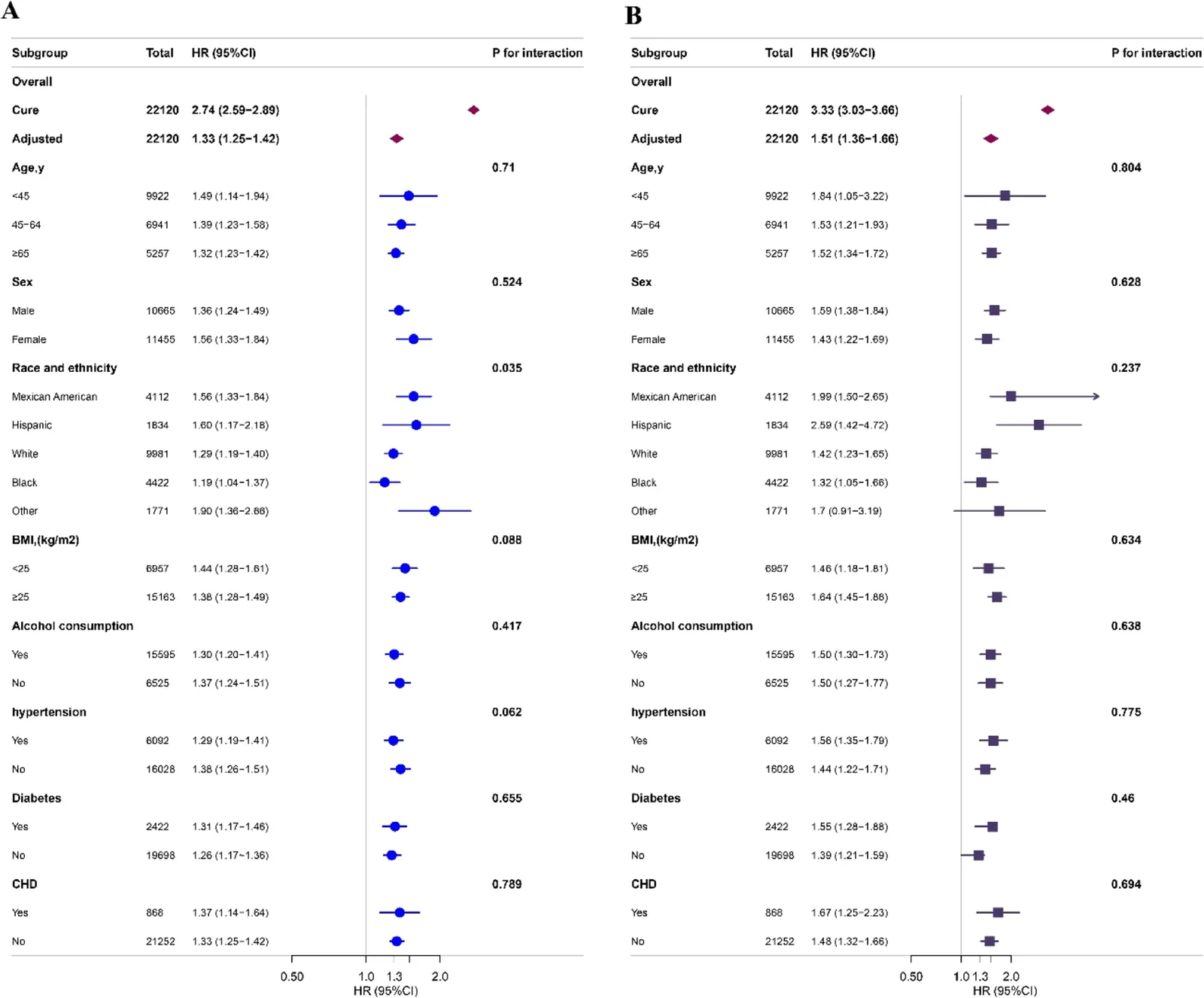
**Forest plots of all-cause and CVD mortality in different subgroups.** Panel A and B display the Hazard Ratios (HRs) and 95% Confidence Intervals (95% CIs) for all-cause and Cardiovascular Disease (CVD) mortality, respectively, across various subgroups, based on Cox proportional hazards models. Multivariable-adjusted models are presented. The adjusted model accounts for age, sex, race and ethnicity, education, PIR, BMI, alcohol consumption, smoke status, hypertension, stroke, Coronary Heart Disease (CHD), creatinine, Blood Urea Nitrogen (BUN), Total Cholesterol (TC), High-Density Lipoprotein Cholesterol (HDL-C), antidiabetic medication use, antihypertensive use, statin use and physical activity. Subgroup analyses were conducted by age, sex, race and ethnicity, alcohol consumption, BMI, hypertension, diabetes, and CHD categories. p-values for interaction indicate the statistical significance of the interaction between GLR and subgroup variables. In Panel A, the association between GLR and all-cause mortality remained significant in most subgroups, with statistically significant interactions for race and ethnicity (p for interaction = 0.035). In Panel B, GLR was also significantly associated with increased cardiovascular mortality, although no significant interaction was observed across subgroups (all p for interaction >0.05).

Stratified analyses across demographic and clinical subgroups showed generally consistent associations between higher GLR and increased risks of all-cause and CVD mortality (Fig. 2). After applying the Bonferroni correction (adjusted α = 0.0031), none of the subgroup interactions retained statistical significance for either all-cause or CVD mortality (all adjusted p > 0.05) (Table S6).

**Fig. 3:**
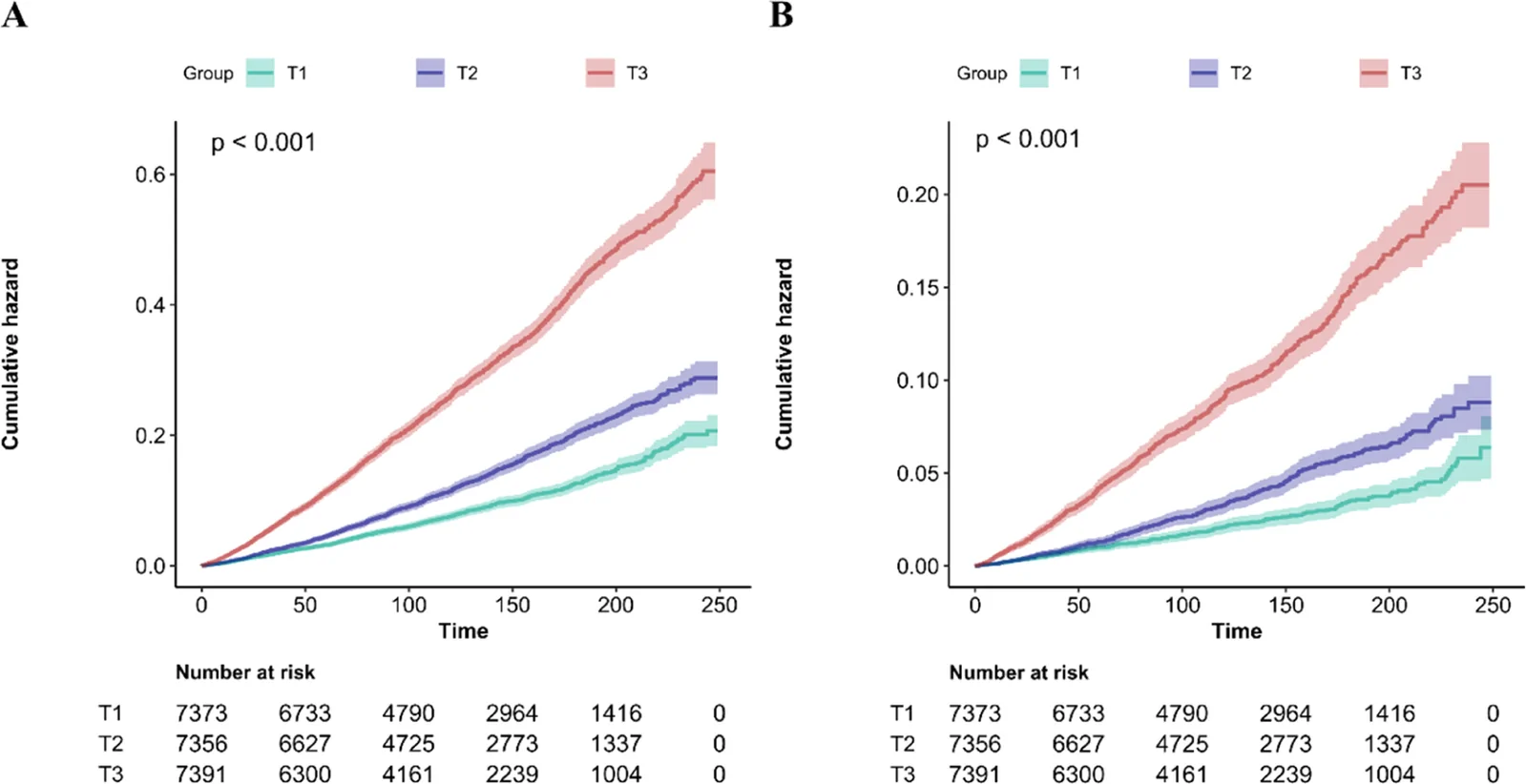
**Cumulative incidence of All-cause mortality and CVD mortality by GLR tertile.** Panels A and B present cumulative hazard curves for all-cause and cardiovascular mortality, respectively, stratified by GLR Tertiles: T1 (lowest), T2 (middle), and T3 (highest). Shaded areas indicate 95% Confidence Intervals. The log-rank test showed statistically significant differences in cumulative hazard among GLR tertile groups (p < 0.0001 for both outcomes).

Kaplan-Meier curves substantiated these findings, showing progressively higher cumulative mortality in higher GLR tertiles for both all-cause and CVD mortality (log-rank p < 0.001, Fig. 3A–B).

### Dose-response relationship between GLR and mortality

**Fig. 4:**
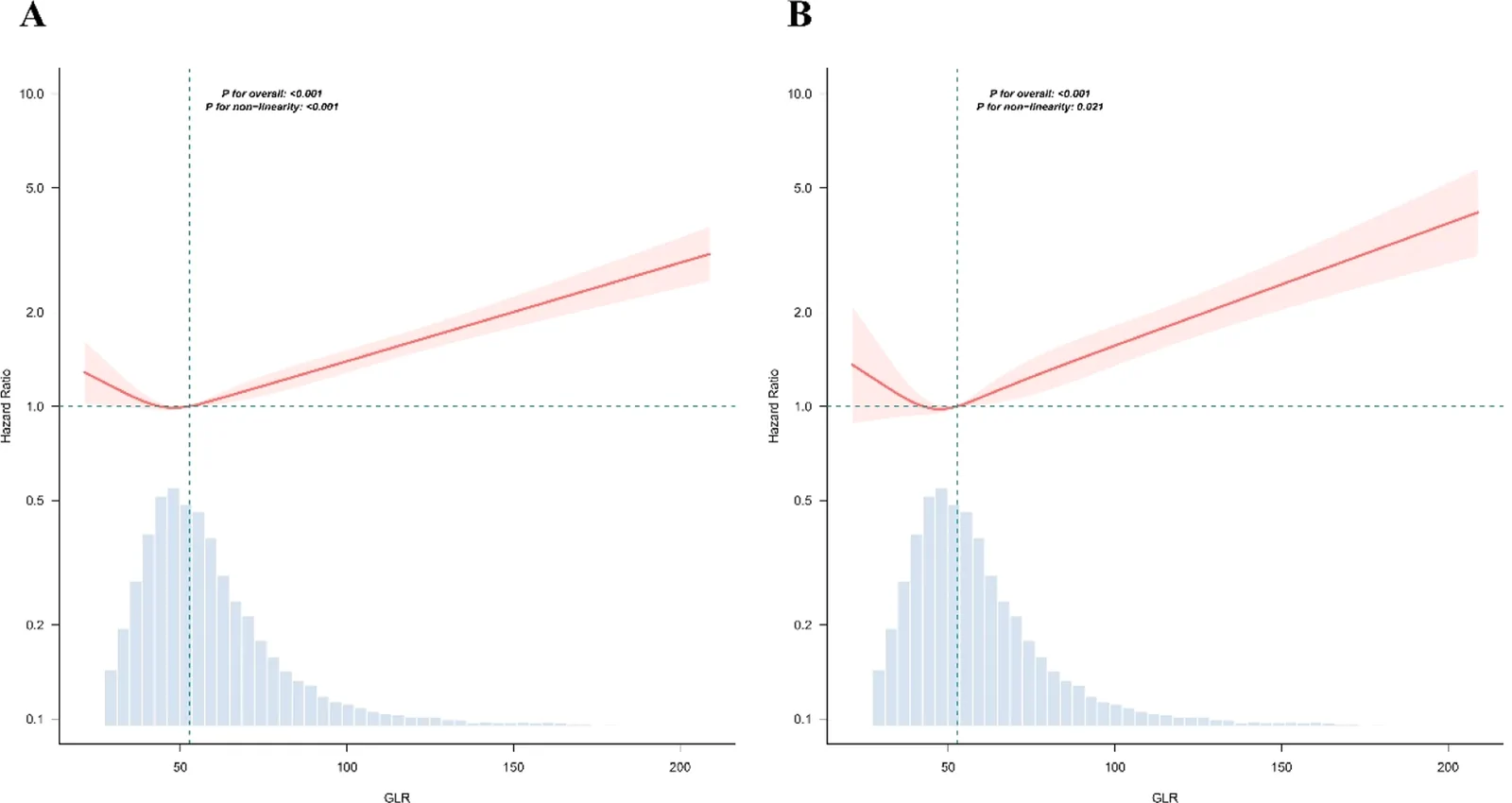
**Restricted cubic spline curves of glucose to lymphocyte ratio and all-cause and CVD mortality.** Panel A: all-cause mortality, p for non-linear < 0.001; Panel B: CVD mortality, p for non-linear = 0.021. The multivariate–adjusted hazard ratios (solid lines) and the 95% Confidence Intervals (dashed lines) derived from restricted cubic spline regression. Knots are positioned at the 5th, 35th, 65th, and 95th percentiles for each of the GLR measures. The horizontal dotted lines represent the odds ratio of 1.0 (Reference point). The Cox regression was adjusted for adjusted factors were age, sex, race and ethnicity, education, PIR, BMI, alcohol consumption, smoke status, hypertension, stroke, Coronary Heart Disease (CHD), cancer, creatinine, Blood Urea Nitrogen (BUN), Total Cholesterol (TC), High-Density Lipoprotein Cholesterol (HDL-C), antidiabetic medication use, antihypertensive use, statin use and physical activity.

Multivariable-adjusted restricted cubic spline studies showed a J-shaped relationship between GLR levels and the risk of all-cause (p for nonlinearity < 0.001). and CVD mortality (p for nonlinearity = 0.021). Mortality risk increased progressively with higher GLR levels, with an inflection zone appearing around GLR values of approximately 46 for all-cause mortality and 49 for CVD mortality (Fig. 4A–B). Below these approximate ranges, the dose-response curves were relatively flat and showed no significant association (p > 0.05). Above these ranges, risk rose steadily (all-cause mortality: HR per unit increase ≈1.007; CVD mortality: HR per unit increase ≈1.009) (Table 3).

**Table 3:** Threshold effect analysis of the relationship between glucose to lymphocyte ratio and All-cause and CVD mortality.

GLR	HR (95% CI)	p-value
**All-cause mortality**		
GLR < 46.74	0.989 (0.977–1.001)	0.0688
GLR ≥ 46.74	1.007 (1.006–1.008)	<0.001
Likelihood Ratio test	–	0.005
**CVD mortality**		
GLR < 48.66	0.988 (0.968–1.007)	0.213
GLR ≥ 48.6656.86	1.009 (1.007–1.012)	<0.001
Likelihood Ratio test	–	0.0236

Hazard Ratios (HRs) and 95% Confidence Interval (95% CI) were derived from multivariable Cox regression models, adjusted for Model 4 (age, sex, race and ethnicity, educational status, PIR, BMI, alcohol consumption, smoke status, hypertension, stroke, coronary heart disease, cancer, creatinine, BUN, TC, HDL-C, antidiabetic medication use, antihypertensive use, statin use and physical activity).

GLR, Glucose to Lymphocyte Ratio; CVD, Cardiovascular Disease; BMI, Body Mass Index; PIR, Poverty-Income Ratio; CHD, Coronary Heart Disease; BUN, Blood Urea Nitrogen; TC, Total Cholesterol; HDL-C, High-Density Lipoprotein Cholesterol.

### Sensitivity analyses

Sensitivity analyses consistent results (Table S7–S12). In fully adjusted Cox models (Model 4), the log_2_-transformed GLR remained associated with all-cause and CVD mortality across multiple scenarios, including the unimputed dataset, different imputation strategies, alternative categorization schemes, analyses incorporating outliers, models excluding extreme values (±3SD), incorporating diabetes as a covariate, and excluding participants with CHD. Additionally, trend tests for both outcomes were statistically significant (p < 0.05). These findings indicate that the associations between GLR and mortality outcomes remain robust across various analytical approaches.

### Laboratory marker comparisons

**Fig. 5:**
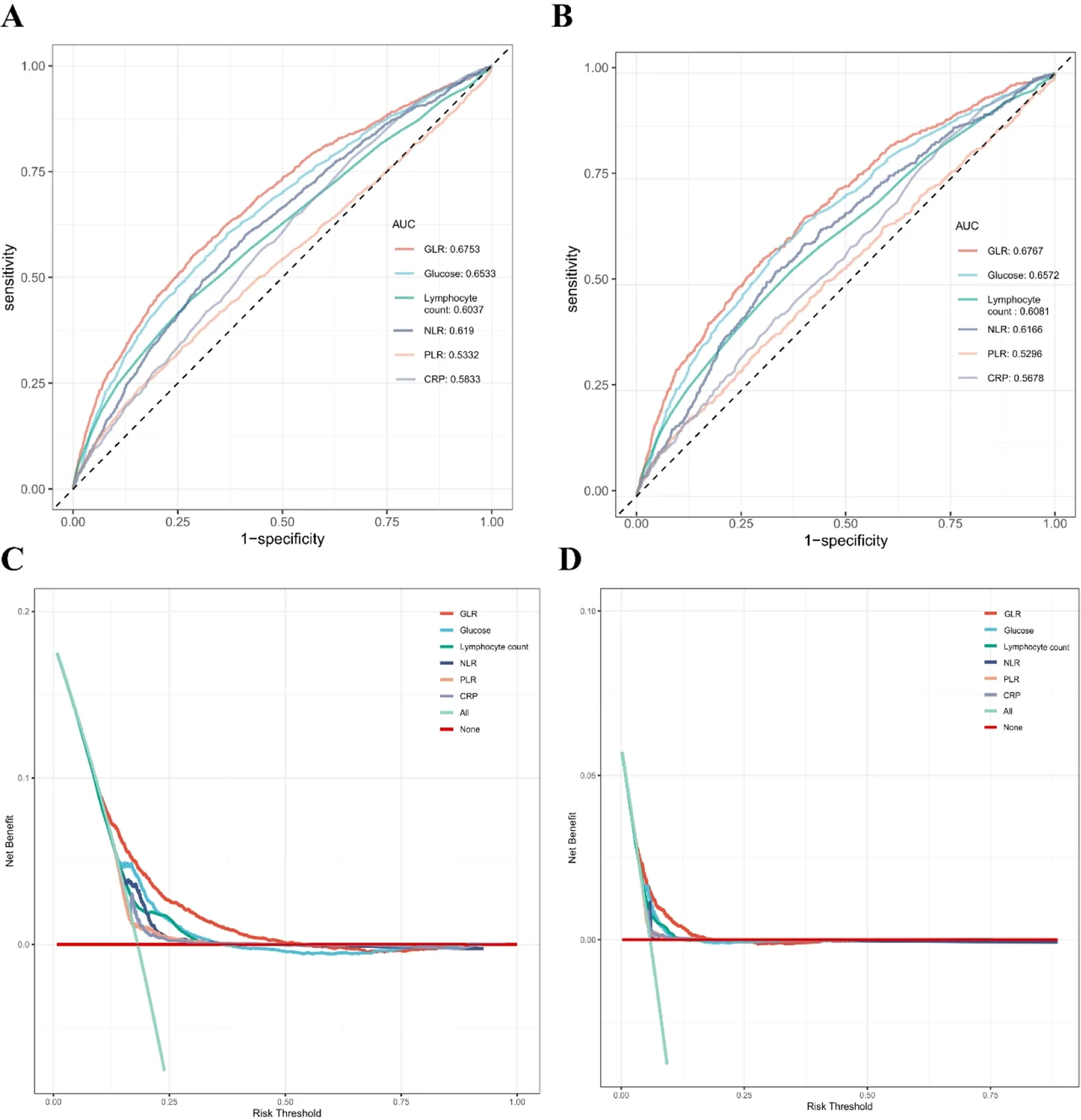
Receiver-Operating Characteristic (ROC) and Decision Curve Analysis (DCA) for all-cause and Cardiovascular Disease (CVD) mortality. Panel A: ROC curves for the association of GLR, glucose, lymphocyte count, Neutrophil-to-Lymphocyte Ratio (NLR), Platelet-to-Lymphocyte Ratio (PLR), and C-Reactive Protein (CRP) with all-cause mortality; AUC values are shown, with GLR ranking highest. Panel B: Same six indices versus CVD mortality; GLR retains the largest AUC. Panel C: DCA of the six markers for all-cause mortality; the GLR curve lies above the “treat-all” and “treat-none” lines across most thresholds, indicating greater net benefit. Panel D: DCA of the six markers for CVD mortality; GLR achieves the highest net benefit, supporting its utility in risk stratification.

In comparative analyses, GLR demonstrated modestly better discrimination for all-cause and CVD mortality compared with glucose, lymphocyte count, NLR, PLR, and CRP (AUC 67.5% and 67.7%, respectively). DCA indicated that GLR provided a net benefit across wider ranged of threshold probabilities (6%–53% for all-cause and 3%–18% for CVD mortality) compared with the other markers, as shown in Fig. 5 (ROC and DCA curves for all markers) and summarized in Table S13 (AUC values and DCA thresholds).

GLR was added to a baseline Framingham risk model to assess incremental predictive value using continuous NRI and IDI. Statistically significant but clinically modest improvements were observed for all-cause mortality (NRI = 0.122, 95% CI 0.083–0.159; IDI = 0.006, 95% CI 0.003–0.010) and CVD mortality (NRI = 0.146, 95% CI 0.086–0.207; IDI = 0.007, 95% CI 0.003–0.012) as detailed in Table S14.

### E-value analysis

E-value analysis was used to assess the potential impact of unmeasured confounding on the associations between GLR and mortality. Point-estimate E-values ranged from 1.69 to 2.38. For all-cause mortality, the E-values were 1.99 (lower CI: 1.81) for the continuous model and 1.69 (1.43) for T3 vs. T1; for CVD mortality, the corresponding values were 2.38 (2.06) and 1.99 (1.51), respectively (Table S15). These magnitudes indicate moderate robustness of the associations to unmeasured confounding.

## Discussion

In this nationally representative U.S. cohort, elevated GLR was independently associated with higher risks of all-cause and cardiovascular mortality, with a J-shaped dose–response pattern. GLR provided modest incremental information beyond traditional Framingham risk factors. The findings suggest that GLR, as a composite metric, might capture intertwined metabolic-immune pathways. It does not replace established risk scores but may complement them. These associations, though modest, are consistent with other metabolic-inflammatory indicators and remain observational, requiring external validation before clinical application.

Previous research has primarily focused on specific patient populations, such as those with cancer,^18^ coronary artery disease,^29^ respiratory diseases,^30^ showing that elevated GLR predicts worse outcomes. In critical care settings, Yang et al.^31^ demonstrated a 73% increased mortality risk among non-traumatic cerebral hemorrhage patients (HR = 1.73, 95% CI 1.18‒2.52), while Liu et al.^32^ reported that higher GLR was associated with increased in-hospital mortality in acute myocardial infarction patients (HR = 1.70, 95% CI 1.24–2.34). Preoperative GLR has also been linked to adverse prognoses in several cancer cohorts.^33^, ^34^, ^35^ Our study extends these findings to the general U.S. adult population, confirming a significant independent association with mortality beyond critically ill or disease-specific groups.

In our analysis using Cox proportional hazards models with restricted cubic splines, elevated GLR was independently associated with higher risks of all-cause and cardiovascular mortality. Compared with its individual components (glucose, lymphocyte count) and inflammatory ratios (NLR, PLR, CRP), GLR provided modest incremental improvements in discrimination and reclassification (AUC, NRI, IDI), suggesting it captures integrated metabolic-immune information beyond individual biomarkers. Unlike platelet-based composite indices such as the Systemic Immune-Inflammation Index (SII), GLR integrates metabolic and immune dimensions, which may offer complementary pathophysiological information. DCA indicated a modest net clinical benefit, though prospective validation is required to determine its practical advantage over established risk scores.

Exploratory subgroup analyses stratified by demographic and clinical characteristics showed broadly consistent associations, with no interactions remaining significant after Bonferroni correction, indicating that GLR's prognostic value is largely stable across population subgroups.

Restricted cubic spline analysis revealed a J-shaped relationship, with inflection around GLR ≈46–49. These inflection points are exploratory and cohort-specific; their generalizability requires validation in independent populations. This may reflect metabolic–immune crosstalk: chronic hyperglycemia impairs lymphocyte function,^36^ lymphopenia disrupts glucose regulation,^37^ and GLR integrates metabolic status and immune suppression.^38^ These mechanisms are plausible hypotheses, not confirmed causally. High glucose may also promote oxidative stress. Threshold analysis aligned with dialysis cohort findings 76% CVD risk increase per GLR unit^39^ and extending Lei et al.’s observation.^40^

Clinically, elevated GLR should be interpreted cautiously. Values above 46–49 were linked to higher mortality, reflecting potential metabolic-immune dysregulation, especially in individuals with metabolic comorbidities or intermediate-risk profiles. Consequently, future prospective studies should focus on validating GLR in intermediate-risk adults or individuals with metabolic syndrome, where it may enhance precision beyond conventional risk scores. GLR is not a standalone diagnostic marker, but may serve as a complementary measure alongside established risk assessments. Serial measurements may provide further insight, but prospective studies are needed to confirm whether changes in GLR are associated with alterations in risk over time.

Our study benefits from a large prospective, population-based cohort with long-term follow-up for all-cause and CVD mortality, providing robust and generalizable findings for the general population.

Several limitations warrant attention. First, as an observational study, associations between GLR and mortality indicate correlation rather than causation. Residual confounding may persist despite adjustment for lifestyle, chronic illnesses, and demographics. Moreover, reverse causality cannot be excluded, as subclinical disease or early pathophysiological changes may influence both GLR levels and subsequent mortality risk. Second, relying on a single baseline glucose measurement may reflect acute rather than chronic glycemic status, which could bias effect estimates toward the null. Third, findings in a general population may not generalize to high-risk clinical groups (spectrum bias). Finally, GLR was measured only once, preventing assessment of its trajectory over time, which may limit understanding of temporal changes and their impact on risk. Multiple sensitivity analyses, however, indicated robustness against potential bias from this single-timepoint measurement. Prospective studies with serial measurements are needed to refine effect estimates, clarify mechanisms, and assess whether GLR meaningfully improves clinical risk prediction.

## Conclusion

In this large, nationally representative cohort, elevated GLR was independently associated with higher risks of all-cause and CVD mortality, with a J-shaped dose-response curve rising above GLR ≈46–49. GLR showed modest but statistically significant additional information beyond traditional risk factors, supported by sensitivity analyses. As a composite marker of metabolic-immune dysregulation, GLR may complement established clinical assessments in primary prevention. Causal inference is limited by the observational design and single baseline measurement. The observed associations demonstrated moderate robustness to unmeasured confounding, as reflected by E-values ranging from 1.69 to 2.38; nevertheless, prospective studies are needed to externally validate these findings, clarify underlying mechanisms, and determine whether serial GLR measurements are associated with changes in risk over time.

## Ethics approval and informed consent

NHANES received approval from the National Center for Health Statistics Research Ethics Review Board (http://www.cdc.gov/nchs/nhanes/about/erb.html). All participants signed written informed consent.

## Consent for publication

Consent was provided through the completion of the survey.

## Funding

The study has no foundation.

## Data availability

Our research is based on public data from the NHANES, all details are from the official website (https://www.cdc.gov/nchs/nhanes/?CDC_AAref_Val) and the National Death Index (https://www.cdc.gov/nchs/data–linkage/mortality.htm). To obtain the application executable files, please contact the author Yongtong He by email heyongtong2024@126.com.

## Conflicts of interest

The authors declare no conflicts of interest.
