# Supplementary material for: Association of the glucose-to-lymphocyte ratio with all-cause and cardiovascular disease mortality in U.S. adults: A population-based cohort study
Source: Clinics (Sao Paulo). 2026 Sep 9;81:101117. doi: 10.1016/j.clinsp.2026.101117 (PMC13573687; doi:10.1016/j.clinsp.2026.101117)
Supplement: Supplementary file 1 [file mmc1.docx]

**CLINICS-D-25-01043**

**Supplement Material**

**Table S1 Missing Data Frequency and Percentage in the Study Cohort.**

**Table S2 Proportional hazards assumption test based on Schoenfeld residuals for GLR with all-cause and CVD mortality.**

**Table S3 Fine-Gray Competing Risk Analysis.**

**Table S4 Model Evaluation Metrics for the Association Between GLR with all-cause and CVD mortality.**

**Table S5 Internal Cross-Validation of Model Performance for GLR Association with All-Cause and Cardiovascular Mortality.**

**Table S6 *P*-value Adjustment in Subgroup Analyzes for GLR-Mortality Associations Using Bonferroni Correction.**

**Table S7 Sensitivity Analysis: GLR-Mortality Associations Under unimputed database.**

**Table S8 Sensitivity Analysis: GLR-Mortality Associations Under Different Imputation Methods.**

**Table S9 Sensitivity Analysis: GLR-Mortality Associations Under using various GLR categorization approaches.**

**Table S10 Sensitivity Analysis of GLR-Mortality Associations With/Without Extreme Value Exclusion.**

**Table S11 Sensitivity Analyzes Including Diabetes as a Covariate for Associations Between GLR and All-Cause and CVD Mortality.**

**Table S12 Sensitivity Analysis of GLR-Mortality Associations Excluding participants with CHD.**

**Table S13 Comparative Discriminative Performance of Biomarkers for Mortality Outcomes.**

**Table S14 Incremental Value of GLR for Mortality Risk Stratification Beyond the Basic Framingham Model.**

**Table S15 E-values for the Associations of GLR With All-Cause and CVD Mortality.**

**Figure legends**

**Fig. S1 Mediation by Systolic Blood Pressure (SBP) of the GLR–mortality link: all-cause and CVD mortality.**

**Fig.S2 Decision Curve Analysis for the four models.**

**Fig. S3 Calibration plots for the four models.**

**Table S1 Missing Data Frequency and Percentage in the Study Cohort.**

| **Variable** | **Missing Frequency** | **Missing percentage(%)** |
| --- | --- | --- |
| Educational status | 873 | 3.907 |
| PIR | 1875 | 8.391 |
| BMI | 366 | 1.638 |
| Alcohol consumption | 2386 | 10.678 |
| Smoke status | 746 | 3.338 |
| Hypertension | 134 | 0.599 |
| Stroke | 862 | 3.857 |
| CHD | 940 | 4.206 |
| Cancer | 859 | 3.844 |
| Diabetes | 13 | 0.058 |
| BUN | 276 | 1.2477 |
| Creatinine | 2494 | 11.2749 |
| TC | 230 | 1.0398 |
| HDL | 230 | 1.0398 |
| Physical activity | 828 | 3.7432 |

Note:

BMI, Body mass index; PIR, Poverty-income ratio; CHD, Coronary heart disease; BUN, Blood urea nitrogen; TC, total cholesterol; HDL-C, High-density lipoprotein cholesterol.

### ****Table S2 Proportional hazards assumption test based on Schoenfeld residuals for GLR with All-cause and CVD mortality.****

| **Variable** | **Chi-square (χ²)** | **df** | ***P* value** |
| --- | --- | --- | --- |
| **All-cause mortality** |  |  |  |
| GLR(log_2_) continuous | 0.849 | 1.0 | 0.357 |
| GLR(log_2_) tertile | 1.11 | 2.0 | 0.574 |
| **CVD mortality** |  |  |  |
| GLR(log_2_) continuous | 0.17 | 1.0 | 0.681 |
| GLR(log_2_) tertile | 1.043 | 2.0 | 0.594 |

Note:

Proportional hazards assumption was assessed using Schoenfeld residuals. For both all-cause and cardiovascular disease (CVD) mortality, the non-significant *P* values (*P* > 0.5) across continuous and tertile-based GLR (log_2_-transformed) indicate no violation of the proportional hazards assumption in the Cox regression models. This supports the validity of the hazard ratios (HRs) reported in the primary analysis.

**Tabel S3 Fine-Gray Competing Risk Analysis.**

| **Variable** | **total** | **No. of events %** | **crude.HR (95%CI)** | ***P*-value** | **adj.HR (95%CI)** | ***P*-value** |
| --- | --- | --- | --- | --- | --- | --- |
| GLR(log_2_) continuous | 22120 | 1161 (5.2) | 2.96 (2.68–3.26) | <0.001 | 1.39 (1.24–1.56) | <0.001 |
| GLR(log_2_) tertile |  |  |  |  |  |  |
| T1 | 7373 | 188 (2.5) | 1(Ref) |  | 1(Ref) |  |
| T2 | 7356 | 294 (4.0) | 1.58 (1.31–1.89) | <0.001 | 1.01 (0.84–1.22) | 0.901 |
| T3 | 7391 | 679 (9.2) | 3.80 (3.23–4.47) | <0.001 | 1.35 (1.14–1.6) | 0.001 |
| *P-*value for trend test |  |  |  | <0.001 |  | <0.001 |

Note: Adjusted for Model 4: age, sex, race and ethnicity, educational status, PIR, BMI, alcohol consumption, smoke status, hypertension, stroke, coronary heart disease, cancer, creatinine, BUN, TC, HDL-C, antidiabetic medication use, antihypertensive use, statin use and physical activity. Ref = Reference group. GLR values are log2 transformed. Using the Fine–Gray subdistribution hazard model to account for the competing risk of non-CVD death, a significant positive association between GLR (both continuous and in tertiles) and cardiovascular mortality risk was confirmed after full adjustment. These findings were consistent with the primary Cox regression results, indicating that the presence of competing risks (non-CVD death) did not materially affect the observed associations. P-value for trend test < 0.001 for both crude and adjusted models.

**Table S4 Model Evaluation Metrics for GLR Association with All-cause and CVD mortality.**

| **Variable** | **C-index** | **Brier** | **AIC** | **BIC** | **R2** | **RMSE** |
| --- | --- | --- | --- | --- | --- | --- |
| **All-cause mortality** |  |  |  |  |  |  |
| Model 1 | 65.85% (64.84% ~ 66.86%) | 0.056 | 67738.95 | 67746.95 | 0.05 | 0.41 |
| Model 2 | 87.94% (87.34% ~ 88.55%) | 0.048 | 61580.65 | 61668.70 | 0.3 | 0.40 |
| Model 3 | 88.72% (88.13% ~ 89.30%) | 0.047 | 61185.79 | 61353.88 | 0.31 | 0.41 |
| Model 4 | 89.45% (88.88% ~ 90.02%) | 0.046 | 60942.54 | 611802.67 | 0.32 | 0.41 |
| **CVD mortality** |  |  |  |  |  |  |
| Model 1 | 67.24% (65.57% ~ 68.91%) | 0.020 | 21473.70 | 21481.71 | 0.04 | 0.23 |
| Model 2 | 86.47% (85.5% ~ 87.43%) | 0.019 | 19192.33 | 19280.38 | 0.19 | 0.23 |
| Model 3 | 87.25% (86.32% ~ 88.18%) | 0.019 | 19043.05 | 19211.14 | 0.20 | 0.23 |
| Model 4 | 87.84% (86.94% ~ 88.75%) | 0.018 | 184948.41 | 19188.54 | 0.21 | 0.23 |

Note:

Model 1: no adjusted covariates.

Model 2: adjusted for age, sex, race and ethnicity, educational status, PIR.

Model 3: adjusted for model 2 plus BMI, alcohol consumption, smoke status, hypertension, stroke, coronary heart disease, cancer.

Model 4: adjusted for model 3 plus creatinine, BUN, TC, HDL-C, antidiabetic medication use, antihypertensive use, statin use and physical activity.

GLR, glucose to lymphocyte ratio; CVD, Cardiovascular disease; BMI, Body mass index; PIR, Poverty-income ratio; CHD, Coronary heart disease; BUN, Blood urea nitrogen; TC, total cholesterol; HDL-C, High-density lipoprotein cholesterol.

C-index: Quantifies discrimination ability (range 50-100%); Brier score: Measures overall model accuracy (lower values preferred); AIC/BIC: Evaluate model fit with penalty for complexity; R²: Represents explained variation; RMSE: Indicates model error magnitude.

Progressive improvement in model fit with increasing adjustment levels. Model 4 demonstrated optimal balance of discrimination and calibration.

**Table S5 Internal Cross-Validation of Model Performance for GLR Association with All-Cause and Cardiovascular Mortality.**

| **Variable** | **AUC (mean)** | **Bier (mean)** |
| --- | --- | --- |
| **All cause mortality** |  |  |
| Model 1 | 0.678 | 0.056 |
| Model 2 | 0.868 | 0.048 |
| Model 3 | 0.876 | 0.047 |
| Model 4 | 0.881 | 0.046 |
| **CVD mortality** |  |  |
| Model 1 | 0.703 | 0.020 |
| Model 2 | 0.901 | 0.018 |
| Model 3 | 0.905 | 0.018 |
| Model 4 | 0.916 | 0.018 |

Note:

Model 1: no adjusted covariates.

Model 2: adjusted for age, sex, race and ethnicity, educational status, PIR.

Model 3: adjusted for model 2 plus BMI, alcohol consumption, smoke status, hypertension, stroke, coronary heart disease, cancer.

Model 4: adjusted for model 3 plus creatinine, BUN, TC, HDL-C, antidiabetic medication use, antihypertensive use, statin use and physical activity.

GLR, glucose to lymphocyte ratio; CVD, Cardiovascular disease; BMI, Body mass index; PIR, Poverty-income ratio; CHD, Coronary heart disease; BUN, Blood urea nitrogen; TC, total cholesterol; HDL-C, High-density lipoprotein cholesterol.

AUC values represent the mean discrimination performance across internal cross-validation folds, ranging from 0.5 (no discrimination) to 1.0 (perfect discrimination). Brier scores reflect mean prediction error (0–1), with lower values indicating better overall accuracy. Both metrics were estimated using k-fold cross-validation to evaluate model generalizability.

For both all-cause and CVD mortality, mean AUCs increased and Brier scores decreased progressively from Model 1 to Model 4, indicating improved discrimination and calibration with increasing model adjustment. The fully adjusted Model 4 achieved the highest mean AUC and lowest Brier score.

**Table S6 *P*-value Adjustment in Subgroup Analyzes for GLR-Mortality Associations Using Bonferroni Correction.**

| **Variable** | **Unadjusted *P*-value** | **Adjusted *P*-value** |
| --- | --- | --- |
| **All cause mortality** |  |  |
| Age-stratified | 0.710 | 1.0 |
| Sex-stratified | 0.524 | 1.0 |
| Race and ethnicity-stratified | 0.035 | 0.28 |
| BMI-stratified | 0.269 | 1.0 |
| Alcohol consumption -stratified | 0.417 | 1.0 |
| Hypertension -stratified | 0.062 | 0.496 |
| Diabetes-stratified | 0.655 | 1.0 |
| CHD-stratified | 0.789 | 1.0 |
| **CVD mortality** |  |  |
| Age-stratified | 0.804 | 1 |
| Sex-stratified | 0.628 | 1 |
| Race and ethnicity-stratified | 0.237 | 1 |
| BMI-stratified | 0.247 | 1 |
| Alcohol consumption-stratified | 0.638 | 1 |
| Hypertension -stratified | 0.775 | 1 |
| Diabetes-stratified | 0.460 | 1 |
| CHD-stratified | 0.694 | 1 |

NOTE:

Bonferroni adjustment applied to control family-wise error rate across 16 subgroup comparisons (8 strata × 2 mortality outcomes). Adjusted significance threshold: α = 0.003125 (0.05/16).

All originally significant subgroup associations became non-significant after adjustment (adjusted *P*> 0.003125). No subgroup demonstrated statistically robust associations after accounting for multiple testing.

**Table S7 Sensitivity Analysis: GLR-Mortality Associations Under unimputed database.**

| **Variable** | **Total** | **Model 1** | | **Model 2** | | **Model 3** | | **Model 4** | |
| --- | --- | --- | --- | --- | --- | --- | --- | --- | --- |
|  |  | **HR(95% CI)** | ***P*-value** | **HR(95% CI)** | ***P*-value** | **HR(95% CI)** | ***P*-value** | **HR(95% CI)** | ***P*-value** |
| All–cause mortality |  |  |  |  |  |  |  |  |  |
| GLR(log_2_) continuous | 22120 | 2.74 (2.59–2.89) | <0.001 | 1.35 (1.27–1.44) | <0.001 | 1.35 (1.26–1.44) | <0.001 | 1.30 (1.20–1.40) | <0.001 |
| GLR(log_2_) tertile |  |  |  |  |  |  |  |  |  |
| T1 | 7373 | 1(Ref) |  | 1(Ref) |  | 1(Ref) |  | 1(Ref) |  |
| T2 | 7356 | 1.50 (1.37–1.66) | <0.001 | 0.97 (0.88–1.08) | 0.578 | 0.99 (0.88–1.10) | 0.838 | 0.96 (0.85–1.09) | 0.549 |
| T3 | 7391 | 3.27 (3.00–3.57) | <0.001 | 1.21 (1.10–1.33) | <0.001 | 1.22 (1.10–1.35) | <0.001 | 1.19 (1.06–1.33) | 0.003 |
| *P*–value for trend test |  |  | <0.001 |  | <0.001 |  | <0.001 |  | 0.001 |
| CVD mortality |  |  |  |  |  |  |  |  |  |
| GLR(log_2_) continuous | 22120 | 3.33 (3.03–3.66) | <0.001 | 1.60 (1.43–1.78) | <0.001 | 1.55 (1.37–1.74) | <0.001 | 1.53 (1.34–1.75) | <0.001 |
| GLR(log_2_) tertile |  |  |  |  |  |  |  |  |  |
| T1 | 7373 | 1(Ref) |  | 1(Ref) |  | 1(Ref) |  | 1(Ref) |  |
| T2 | 7356 | 1.61 (1.34–1.93) | <0.001 | 1.00 (0.82–1.21) | 0.992 | 0.96 (0.78–1.18) | 0.715 | 0.97 (0.77–1.22) | 0.815 |
| T3 | 7391 | 4.16 (3.54–4.89) | <0.001 | 1.41 (1.18–1.68) | <0.001 | 1.32 (1.09–1.59) | 0.004 | 1.31 (1.06–1.61) | 0.013 |
| *P*–value for trend test |  |  | <0.001 |  | <0.001 |  | <0.001 |  | 0.001 |

Model 1: no adjusted covariates.

Model 2: adjusted for age, sex, race and ethnicity, educational status, PIR.

Model 3: adjusted for model 2 plus BMI, alcohol consumption, smoke status, hypertension, stroke, coronary heart disease, cancer.

Model 4: adjusted for model 3 plus creatinine, BUN, TG, HDL-C, antidiabetic medication use, antihypertensive use, stain use and physical activity.

GLR, glucose to lymphocyte ratio; CVD, Cardiovascular disease; BMI, Body mass index; PIR, Poverty-income ration; CHD, Coronary heart disease; BUN, Blood urea nitrogen; TC, total cholesterol; HDL-C, High-density lipoprotein cholesterol.

**Table S8 Sensitivity Analysis: GLR-Mortality Associations Under Different Imputation Methods.**

| **Variable** | **Total** | **Model 1** | | **Model 2** | | **Model 3** | | **Model 4** | |
| --- | --- | --- | --- | --- | --- | --- | --- | --- | --- |
|  |  | **HR(95% CI)** | ***P*-value** | **HR(95% CI)** | ***P*-value** | **HR(95% CI)** | ***P*-value** | **HR (95% CI)** | **P-value** |
| **Simple Substitution** | | | | | | | | | |
| All–cause mortality |  |  |  |  |  |  |  |  |  |
| GLR(log_2_) continuous | 22120 | 2.74 (2.59–2.89) | <0.001 | 1.33 (1.25–1.42) | <0.001 | 1.34 (1.26–1.42) | <0.001 | 1.28 (1.20–1.36) | <0.001 |
| GLR(log_2_) tertile |  |  |  |  |  |  |  |  |  |
| T1 | 7373 | 1(Ref) |  | 1(Ref) |  | Ref |  |  |  |
| T2 | 7356 | 1.50 (1.37–1.66) | <0.001 | 0.92 (0.84–1.02) | 0.115 | 0.95 (0.86–1.04) | 0.279 | 0.94 (0.85–1.04) | 0.223 |
| T3 | 7391 | 3.27 (3.00–3.57) | <0.001 | 1.17 (1.07–1.28) | 0.001 | 1.20 (1.09–1.31) | <0.001 | 1.15 (1.05–1.26) | 0.002 |
| *P*–value for trend test |  |  | <0.001 |  | <0.001 |  | <0.001 |  | <0.001 |
| **CVD mortality** |  |  |  |  |  |  |  |  |  |
| GLR(log_2_) continuous | 22120 | 3.33 (3.03–3.66) | <0.001 | 1.59 (1.43–1.77) | <0.001 | 1.56 (1.41–1.73) | <0.001 | 1.45 (1.30–1.61) | <0.001 |
| GLR(log_2_) tertile |  |  |  |  |  |  |  |  |  |
| T1 | 7373 | 1(Ref) |  | 1(Ref) |  | 1(Ref) |  |  |  |
| T2 | 7356 | 1.61 (1.34–1.93) | <0.001 | 0.94 (0.78–1.13) | 0.538 | 0.95 (0.79–1.15) | 0.607 | 0.94 (0.78–1.14) | 0.538 |
| T3 | 7391 | 4.16 (3.54–4.89) | <0.001 | 1.37 (1.16–1.61) | <0.001 | 1.37 (1.16–1.62) | <0.001 | 1.30 (1.09–1.53) | 0.003 |
| *P*–value for trend test |  |  | <0.001 |  | <0.001 |  | <0.001 |  | <0.001 |
| **Dummy Variable Adjustment** | | | | | | | | | |
| **All–cause mortality** | | | | | | | | | |
| GLR(log_2_) continuous | 22120 | 2.74 (2.59–2.89) | <0.001 | 1.33 (1.25–1.42) | <0.001 | 1.34 (1.26–1.42) | <0.001 | 1.28 (1.20–1.36) | <0.001 |
| GLR(log_2_) tertile |  |  |  |  |  |  |  |  |  |
| T1 | 7373 | 1(Ref) |  | 1(Ref) |  | 1(Ref) |  | 1(Ref) |  |
| T2 | 7356 | 1.50 (1.37–1.66) | <0.001 | 0.92 (0.84–1.02) | 0.115 | 0.95 (0.86–1.04) | 0.279 | 0.94 (0.85–1.04) | 0.233 |
| T3 | 7391 | 3.27 (3.00–3.57) | <0.001 | 1.17 (1.07–1.28) | 0.001 | 1.20 (1.09–1.31) | <0.001 | 1.15 (1.05–1.26) | 0.002 |
| *P*–value for trend test |  |  | <0.001 |  | <0.001 |  | <0.001 |  | <0.001 |
| **CVD mortality** |  |  |  |  |  |  |  |  |  |
| GLR(log_2_) continuous | 22120 | 3.33 (3.03~3.66) | <0.001 | 1.59 (1.43–1.77) | <0.001 | 1.56 (1.41–1.73) | <0.001 | 1.45 (1.3–1.61) | <0.001 |
| GLR(log2) tertile |  |  |  |  |  |  |  |  |  |
| T1 | 7373 | 1(Ref) |  | 1(Ref) |  | 1(Ref) |  | 1(Ref) |  |
| T2 | 7356 | 1.61 (1.34–1.93) | <0.001 | 0.94 (0.78–1.13) | 0.538 | 0.95 (0.79–1.15) | 0.607 | 0.94 (0.78–1.14) | 0.544 |
| T3 | 7391 | 4.16 (3.54–4.89) | <0.001 | 1.37 (1.16–1.61) | <0.001 | 1.37 (1.16–1.62) | <0.001 | 1.3 (1.10–1.54) | 0.002 |
| *P-*value for trend test |  |  | <0.001 |  | <0.001 |  | <0.001 |  | <0.001 |
| **Decision-Tree-Based Imputation** | | | | | | | | | |
| **All–cause mortality** | | | | | | | | | |
| GLR(log_2_) continuous | 22120 | 2.74 (2.59–2.89) | <0.001 | 1.34 (1.26–1.42) | <0.001 | 1.36 (1.28–1.45) | <0.001 | 1.31 (1.23–1.39) | <0.001 |
| GLR(log_2_) tertile |  |  |  |  |  |  |  |  |  |
| T1 | 7373 | 1(Ref) |  | 1(Ref) |  | 1(Ref) |  | 1(Ref) |  |
| T2 | 7356 | 1.50 (1.37–1.66) | <0.001 | 0.93 (0.84–1.03) | 0.154 | 0.97 (0.88–1.07) | 0.519 | 0.96 (0.87–1.06) | 0.391 |
| T3 | 7391 | 3.27 (3.00–3.57) | <0.001 | 1.17 (1.07–1.28) | <0.001 | 1.21 (1.11–1.33) | <0.001 | 1.17 (1.06–1.28) | 0.001 |
| *P-*value for trend test |  | 1.87 (1.79–1.95) | <0.001 |  | <0.001 |  | <0.001 |  | <0.001 |
| **CVD mortality** |  |  |  |  |  |  |  |  |  |
| GLR(log_2_) continuous | 22120 | 3.33 (3.03~3.66) | <0.001 | 1.59 (1.43~1.77) | <0.001 | 1.57 (1.42–1.75) | <0.001 | 1.49 (1.33–1.66) | <0.001 |
| GLR(log_2_) tertile |  |  |  |  |  |  |  |  |  |
| T1 | 7373 | 1(Ref) |  | 1(Ref) |  | 1(Ref) |  | 1(Ref) |  |
| T2 | 7356 | 1.61 (1.34–1.93) | <0.001 | 0.95 (0.79–1.14) | 0.575 | 0.96 (0.80–1.15) | 0.663 | 0.95 (0.79–1.15) | 0.615 |
| T3 | 7391 | 4.16 (3.54–4.89) | <0.001 | 1.37 (1.16–1.62) | <0.001 | 1.36 (1.15–1.61) | <0.001 | 1.30 (1.09–1.54) | 0.002 |
| *P-*value for trend test |  |  | <0.001 |  | <0.001 |  | <0.001 |  | <0.001 |

Model 1: no adjusted covariates.

Model 2: adjusted for age, sex, race and ethnicity, educational status, PIR.

Model 3: adjusted for model 2 plus BMI, alcohol consumption, smoke status, hypertension, stroke, coronary heart disease, cancer.

Model 4: adjusted for model 3 plus creatinine, BUN, TG, HDL-C, antidiabetic medication use, antihypertensive use, stain use and physical activity.

GLR, glucose to lymphocyte ratio; CVD, Cardiovascular disease; BMI, Body mass index; PIR, Poverty-income ration; CHD, Coronary heart disease; BUN, Blood urea nitrogen; TC, total cholesterol; HDL-C, High-density lipoprotein cholesterol.

**Table S9 Sensitivity Analysis: GLR-Mortality Associations Under using various GLR categorization approaches.**

| **Variable** | **Total** | **Model 1** | | **Model 2** | | **Model 3** | | **Model 4** | |
| --- | --- | --- | --- | --- | --- | --- | --- | --- | --- |
|  |  | **HR(95% CI)** | ***P*-value** | **HR(95% CI)** | ***P*-value** | **HR(95% CI)** | ***P*-value** | **HR (95% CI)** | ***P*-value** |
| **Quartiles** | | | | | | | | | |
| **All–cause mortality** | | | | | | | | | |
| GLR(log_2_) continuous | 22120 | 2.74 (2.59–2.89) | <0.001 | 1.35 (1.27–1.43) | <0.001 | 1.38 (1.30–1.47) | <0.001 | 1.33 (1.25–1.42) | <0.001 |
| GLR(log_2_) quartile |  |  |  |  |  |  |  |  |  |
| Q1 | 5529 | 1(Ref) |  | 1(Ref) |  | 1(Ref) |  | 1(Ref) |  |
| Q2 | 5531 | 0.92 (0.82–1.03) | 0.154 | 0.92 (0.82–1.03) | 0.154 | 1.00 (0.89–1.12) | 0.961 | 1.00 (0.89–1.13) | 0.974 |
| Q3 | 5529 | 0.90 (0.81–1.01) | 0.062 | 0.90 (0.81–1.01) | 0.062 | 0.97 (0.87–1.08) | 0.593 | 0.97 (0.87–1.08) | 0.6 |
| Q4 | 5531 | 1.24 (1.12–1.38) | <0.001 | 1.24 (1.12–1.38) | <0.001 | 1.35 (1.21–1.49) | <0.001 | 1.31 (1.18–1.45) | <0.001 |
| *P-*value for trend test |  |  | <0.001 |  | <0.001 |  | <0.001 |  | <0.001 |
| **CVD mortality** |  |  |  |  |  |  |  |  |  |
| GLR(log_2_) continuous | 22120 | 3.33 (3.03–3.66) | <0.001 | 1.60 (1.44–1.78) | <0.001 | 1.59 (1.43–1.77) | <0.001 | 1.51 (1.36–1.68) | <0.001 |
| GLR(log_2_) quartile |  |  |  |  |  |  |  |  |  |
| Q1 | 5529 | 1(Ref) |  | 1(Ref) |  | 1(Ref) |  | 1(Ref) |  |
| Q2 | 5531 | 1.27 (1.01–1.58) | 0.037 | 0.94 (0.75–1.18) | 0.598 | 0.99 (0.79–1.23) | 0.91 | 0.99 (0.8–1.24) | 0.962 |
| Q3 | 5529 | 2.01 (1.64–2.47) | <0.001 | 0.99 (0.80–1.21) | 0.909 | 1.02 (0.83–1.26) | 0.825 | 1.04 (0.84–1.28) | 0.732 |
| Q4 | 5531 | 4.80 (3.99–5.78) | <0.001 | 1.50 (1.24–1.81) | <0.001 | 1.53 (1.26–1.85) | <0.001 | 1.47 (1.22–1.79) | <0.001 |
| *P-*value for trend test |  |  | <0.001 |  | <0.001 |  | <0.001 |  | <0.001 |
| **Quintiles** | | | | | | | | | |
| **All–cause mortality** | | | | | | | | | |
| GLR(log_2_) continuous | 22120 | 2.74 (2.59–2.89) | <0.001 | 1.35 (1.27–1.43) | <0.001 | 1.38 (1.30–1.47) | <0.001 | 1.33 (1.25–1.42) | <0.001 |
| GLR(log_2_) quintles |  |  |  |  |  |  |  |  |  |
| Q1 | 4340 | 1(Ref) |  | 1(Ref) |  | 1(Ref) |  | 1(Ref) |  |
| Q2 | 4504 | 1.04 (0.91–1.18) | 0.609 | 0.86 (0.75–0.99) | 0.034 | 0.95 (0.83–1.09) | 0.494 | 0.86 (0.75–0.99) | 0.034 |
| Q3 | 4427 | 1.52 (1.34–1.72) | <0.001 | 0.90 (0.79–1.02) | 0.099 | 0.99 (0.87–1.12) | 0.817 | 0.90 (0.79–1.02) | 0.099 |
| Q4 | 4425 | 2.03 (1.8–2.28) | <0.001 | 0.87 (0.77–0.98) | 0.022 | 0.96 (0.85–1.08) | 0.463 | 0.87 (0.77–0.98) | 0.022 |
| Q5 | 4424 | 3.97 (3.55–4.43) | <0.001 | 1.26 (1.12–1.41) | <0.001 | 1.39 (1.24–1.56) | <0.001 | 1.26 (1.12–1.41) | <0.001 |
| *P-*value for trend test |  |  | <0.001 |  | <0.001 |  | <0.001 |  | <0.001 |
| **CVD mortality** |  |  |  |  |  |  |  |  |  |
| GLR(log_2_) continuous | 22120 | 3.33 (3.03–3.66) | <0.001 | 1.60 (1.44–1.78) | <0.001 | 1.59 (1.43–1.77) | <0.001 | 1.51 (1.36–1.68) | <0.001 |
| GLR(log_2_) quintles |  |  |  |  |  |  |  |  |  |
| Q1 | 4340 | 1(Ref) |  | 1(Ref) |  | 1(Ref) |  | 1(Ref) |  |
| Q2 | 4504 | 0.95 (0.73–1.23) | 0.689 | 0.95 (0.73–1.23) | 0.689 | 0.95 (0.73–1.23) | 0.689 | 0.95 (0.73–1.23) | 0.689 |
| Q3 | 4427 | 1.07 (0.84–1.35) | 0.6 | 1.07 (0.84–1.35) | 0.600 | 1.07 (0.84–1.35) | 0.6 | 1.07 (0.84–1.35) | 0.6 |
| Q4 | 4425 | 1.03 (0.82–1.29) | 0.826 | 1.03 (0.82–1.29) | 0.826 | 1.03 (0.82–1.29) | 0.826 | 1.03 (0.82–1.29) | 0.826 |
| Q5 | 4424 | 1.52 (1.23–1.89) | <0.001 | 1.52 (1.23–1.89) | <0.001 | 1.52 (1.23–1.89) | <0.001 | 1.52 (1.23–1.89) | <0.001 |
| *P-*value for trend test |  |  | <0.001 |  | <0.001 |  | <0.001 |  | <0.001 |

Model 1: no adjusted covariates.

Model 2: adjusted for age, sex, race and ethnicity, educational status, PIR.

Model 3: adjusted for model 2 plus BMI, alcohol consumption, smoke status, hypertension, stroke, coronary heart disease, cancer.

Model 4: adjusted for model 3 plus creatinine, BUN, TG, HDL-C, antidiabetic medication use, antihypertensive use, stain use and physical activity.

GLR, glucose to lymphocyte ratio; CVD, Cardiovascular disease; BMI, Body mass index; PIR, Poverty-income ration; CHD, Coronary heart disease; BUN, Blood urea nitrogen; TC, total cholesterol; HDL-C, High-density lipoprotein cholesterol.

**Table S10 Sensitivity Analysis of GLR-Mortality Associations With/Without Extreme Value Exclusion.**

| **Variable** | **Total** | **Model 1** | | **Model 2** | | **Model 3** | | **Model 4** | |
| --- | --- | --- | --- | --- | --- | --- | --- | --- | --- |
|  |  | **HR(95% CI)** | ***P*-value** | **HR(95% CI)** | ***P*-value** | **HR(95% CI)** | ***P*-value** | **HR(95% CI)** | ***P*-value** |
| **Unsorted outliers** | | | | | | | | | |
| **All–cause mortality** |  |  |  |  |  |  |  |  |  |
| GLR(log_2_) continuous | 22344 | 2.39 (2.28–2.51) | <0.001 | 1.31 (1.24–1.39) | <0.001 | 1.31 (1.23–1.39) | <0.001 | 1.29 (1.21–1.38) | <0.001 |
| GLR(log_2_) tertile |  |  |  |  |  |  |  |  |  |
| T1 | 7444 | 1(Ref) |  | 1(Ref) |  | 1(Ref) |  | 1(Ref) |  |
| T2 | 7439 | 1.45 (1.32–1.60) | <0.001 | 0.95 (0.86–1.06) | 0.352 | 0.97 (0.87–1.08) | 0.573 | 0.95 (0.85–1.07) | 0.369 |
| T3 | 7461 | 3.22 (2.96–3.51) | <0.001 | 1.21 (1.10–1.33) | <0.001 | 1.22 (1.10–1.35) | <0.001 | 1.19 (1.07–1.33) | 0.002 |
| *P*–value for trend test |  |  | <0.001 |  | <0.001 |  | <0.001 |  | <0.001 |
| **CVD mortality** |  |  |  |  |  |  |  |  |  |
| GLR(log_2_) continuous | 22344 | 2.71 (2.5–2.93) | <0.001 | 1.47 (1.33–1.63) | <0.001 | 1.43 (1.28–1.59) | <0.001 | 1.44 (1.27–1.62) | <0.001 |
| GLR(log_2_) tertile |  |  |  |  |  |  |  |  |  |
| T1 | 7444 | 1(Ref) |  | 1(Ref) |  | 1(Ref) |  | 1(Ref) |  |
| T2 | 7439 | 1.54 (1.29–1.85) | <0.001 | 0.98 (0.81–1.18) | 0.798 | 0.94 (0.77–1.16) | 0.577 | 0.97 (0.77–1.21) | 0.764 |
| T3 | 7461 | 4.00 (3.41–4.68) | <0.001 | 1.39 (1.17–1.64) | <0.001 | 1.30(1.08–1.56) | 0.005 | 1.31 (1.07–1.62) | 0.010 |
| *P*–value for trend test |  |  | <0.001 |  | <0.001 |  | <0.001 |  | 0.001 |
| **Excluding extreme values defined by the ±3 SD rule** | | | | | | | | | |
| **All–cause mortality** |  |  |  |  |  |  |  |  |  |
| GLR(log_2_) continuous | 21941 | 2.56 (2.4–2.72) | <0.001 | 1.17 (1.09–1.25) | <0.001 | 1.31 (1.15–1.49) | <0.001 | 1.16 (1.07–1.25) | <0.001 |
| GLR(log_2_) tertile |  |  |  |  |  |  |  |  |  |
| T1 | 7313 | 1(Ref) |  | 1(Ref) |  | 1(Ref) |  | 1(Ref) |  |
| T2 | 7300 | 1.44 (1.31–1.59) | <0.001 | 0.96 (0.86–1.06) | 0.426 | 0.95 (0.77–1.16) | 0.607 | 0.97 (0.86–1.10) | 0.654 |
| T3 | 7328 | 2.97 (2.72–3.24) | <0.001 | 1.13 (1.03–1.25) | 0.011 | 1.21 (1.00–1.46) | 0.046 | 1.1 3(1.01–1.27) | 0.038 |
| *P*–value for trend test |  |  | <0.001 |  | 0.001 |  | 0.008 |  | 0.011 |
| **CVD mortality** |  |  |  |  |  |  |  |  |  |
| GLR(log_2_) continuous | 21941 | 3.15 (2.81–3.53) | <0.001 | 1.37 (1.21–1.55) | <0.001 | 1.31 (1.15–1.49) | <0.001 | 1.29 (1.11–1.50) | 0.001 |
| GLR(log_2_) tertile |  |  |  |  |  |  |  |  |  |
| T1 | 7313 | 1(Ref) |  | 1(Ref) |  | 1(Ref) |  | 1(Ref) |  |
| T2 | 7300 | 1.52 (1.27–1.83) | <0.001 | 0.97 (0.80–1.17) | 0.743 | 0.95 (0.77–1.16) | 0.607 | 0.97(0.78–1.23) | 0.859 |
| T3 | 7328 | 3.67 (3.12–4.31) | <0.001 | 1.28 (1.08–1.53) | 0.005 | 1.21 (1.00–1.46) | 0.046 | 1.21 (0.98–1.49) | 0.038 |
| *P*–value for trend test |  |  | <0.001 |  | <0.001 |  | 0.008 |  | 0.029 |

Model 1: no adjusted covariates.

Model 2: adjusted for age, sex, race and ethnicity, educational status, PIR.

Model 3: adjusted for model 2 plus BMI, alcohol consumption, smoke status, hypertension, stroke, coronary heart disease, cancer.

Model 4: adjusted for model 3 plus creatinine, BUN, TG, HDL-C, antidiabetic medication use, antihypertensive use, stain use and physical activity.

GLR, glucose to lymphocyte ratio; CVD, Cardiovascular disease; BMI, Body mass index; PIR, Poverty-income ration; CHD, Coronary heart disease; BUN, Blood urea nitrogen; TC, total cholesterol; HDL-C, High-density lipoprotein cholesterol.

**Table S11 Sensitivity Analyzes Including Diabetes as a Covariate for Associations Between GLR and All-Cause and CVD Mortality.**

| **Variable** | **Total** | **Model 1** | | **Model 2** | | **Model 3** | | **Model 4** | |
| --- | --- | --- | --- | --- | --- | --- | --- | --- | --- |
|  |  | **HR(95% CI)** | ***P*-value** | **HR(95% CI)** | ***P*-value** | **HR(95% CI)** | ***P*-value** | **HR (95% CI)** | ***P*-value** |
| **All-cause mortality** |  |  |  |  |  |  |  |  |  |
| GLR(log_2_) continuous | 22120 | 2.74 (2.59–2.89) | <0.001 | 1.35 (1.27–1.43) | <0.001 | 1.26 (1.18–1.36) | <0.001 | 1.25 (1.16–1.35) | <0.001 |
| GLR(log_2_) tertile |  |  |  |  |  |  |  |  |  |
| T1 | 7373 | 1(Ref) |  | 1(Ref) |  | Ref |  |  |  |
| T2 | 7356 | 1.50 (1.37–1.66) | <0.001 | 0.94 (0.85–1.04) | 0.240 | 0.98 (0.88–1.10) | 0.725 | 0.95 (0.84–1.07) | 0.414 |
| T3 | 7391 | 3.27 (3.00–3.57) | <0.001 | 1.19 (1.09–1.30) | <0.001 | 1.16 (1.04–1.28) | 0.006 | 1.14 (1.02–1.28) | 0.025 |
| *P*-value for trend test |  |  | <0.001 |  | <0.001 |  | 0.001 |  | <0.001 |
| CVD mortality |  |  |  |  |  |  |  |  |  |
| GLR(log_2_) continuous | 22120 | 3.33 (3.03–3.66) | <0.001 | 1.60 (1.44–1.78) | <0.001 | 1.45 (1.28–1.64) | <0.001 | 1.48 (1.29–1.70) | <0.001 |
| GLR(log_2_) tertile |  |  |  |  |  |  |  |  |  |
| T1 | 7373 | 1(Ref) |  | 1(Ref) |  | 1(Ref) |  |  |  |
| T2 | 7356 | 1.61 (1.34–1.93) | <0.001 | 0.96 (0.8–1.16) | 0.698 | 0.96 (0.78–11.14.17) | 0.67 | 0.97 (0.77–1.22) | 0.801 |
| T3 | 7391 | 4.16 (3.54–4.89) | <0.001 | 1.39 (1.18–1.64) | <0.001 | 1.25 (1.04–1.51) | 0.019 | 1.28 (1.04–1.58) | 0.022 |
| *P*-value for trend test |  |  | <0.001 |  | <0.001 |  | 0.002 |  | 0.003 |

Model 1: no adjusted covariates.

Model 2: adjusted for age, sex, race and ethnicity, educational status, PIR.

Model 3: adjusted for model 2 plus BMI, alcohol consumption, smoke status, hypertension, stroke, coronary heart disease, cancer, diabetes.

Model 4: adjusted for model 3 plus creatinine, BUN, TG, HDL-C, antidiabetic medication use, antihypertensive use, stain use and physical activity.

GLR, glucose to lymphocyte ratio; CVD, Cardiovascular disease; BMI, Body mass index; PIR, Poverty-income ration; CHD, Coronary heart disease; BUN, Blood urea nitrogen; TC, total cholesterol; HDL-C, High-density lipoprotein cholesterol.

**Table S12 Sensitivity Analysis of GLR-Mortality Associations Excluding participants with CHD.**

| **Variable** | **Total** | **Model 1** | | **Model 2** | | **Model 3** | | **Model 4** | |
| --- | --- | --- | --- | --- | --- | --- | --- | --- | --- |
|  |  | **HR(95% CI)** | ***P*-value** | **HR(95% CI)** | ***P*-value** | **HR(95% CI)** | ***P*-value** | **HR(95% CI)** | ***P*-value** |
| **All–cause mortality** |  |  |  |  |  |  |  |  |  |
| GLR(log_2_) continuous | 21252 | 2.72 (2.56–2.88) | <0.001 | 1.39 (1.3–1.48) | <0.001 | 1.43 (1.34–1.53) | <0.001 | 1.36 (1.28–1.46) | <0.001 |
| GLR(log_2_) tertile |  |  |  |  |  |  |  |  |  |
| T1 | 7248 | 1(Ref) |  | 1(Ref) |  | 1(Ref) |  | 1(Ref) |  |
| T2 | 7113 | 1.50 (1.36–1.66) | <0.001 | 0.98 (0.89–1.09) | 0.75 | 1.04 (0.94–1.16) | 0.418 | 1.02 (0.92–1.13) | 0.688 |
| T3 | 6891 | 3.21 (2.93–3.52) | <0.001 | 1.26 (1.15~1.39) | <0.001 | 1.34 (1.22–1.47) | <0.001 | 1.27 (1.16–1.40) | <0.001 |
| *P*–value for trend test |  |  | <0.001 |  | <0.001 |  | <0.001 |  | <0.001 |
| **CVD mortality** |  |  |  |  |  |  |  |  |  |
| GLR(log_2_) continuous | 21941 | 3.28 (2.95–3.64) | <0.001 | 1.63 (1.46–1.83) | <0.001 | 1.65 (1.47–1.85) | <0.001 | 1.54 (1.37–1.73) | <0.001 |
| GLR(log_2_) tertile |  |  |  |  |  |  |  |  |  |
| T1 | 7313 | 1(Ref) |  | 1(Ref) |  | 1(Ref) |  | 1(Ref) |  |
| T2 | 7300 | 1.60 (1.32–1.94) | <0.001 | 1.01 (0.83–.22) | 0.952 | 1.04 (0.85–1.27) | 0.695 | 1.02 (0.83–1.24) | 0.87 |
| T3 | 7328 | 4.02 (3.38–4.77) | <0.001 | 1.47 (1.23–1.75) | <0.001 | 1.51 (1.26–1.8) | <0.001 | 1.42 (1.19–1.70) | <0.001 |
| *P*–value for trend test |  |  | <0.001 |  | <0.001 |  | <0.001 |  | <0.001 |

Model 1: no adjusted covariates.

Model 2: adjusted for age, sex, race and ethnicity, educational status, PIR.

Model 3: adjusted for model 2 plus BMI, alcohol consumption, smoke status, hypertension, stroke, coronary heart disease, cancer.

Model 4: adjusted for model 3 plus creatinine, BUN, TG, HDL-C, antidiabetic medication use, antihypertensive use, stain use and physical activity.

GLR, glucose to lymphocyte ratio; CVD, Cardiovascular disease; BMI, Body mass index; PIR, Poverty-income ration; CHD, Coronary heart disease; BUN, Blood urea nitrogen; TC, total cholesterol; HDL-C, High-density lipoprotein cholesterol.

**Table S13 Comparative Discriminative Performance of Biomarkers for Mortality Outcomes.**

| **Variable** | **AUC (95% CI)** | **DCA Thresholds** |
| --- | --- | --- |
| **All-cause mortality** |  |  |
| GLR | 67.53% (66.11–68.95%) | 6–53% |
| Glucose | 65.33% (63.89– 66.77%) | 6-35% |
| Lymphocyte count | 60.37% (58.85 – 61.89%) | 14-34% |
| NLR | 63.24% (61.78–64.70%) | 6–50% |
| PLR | 53.32% (51.76–54.88%) | 6–56% |
| CRP | 58.33% (56.91–59.74%) | 6–50% |
| **CVD mortality** |  |  |
| GLR | 67.67% (65.38–69.96%) | 3–18% |
| Glucose | 65.72% (63.39– 68.04%) | 4-13% |
| Lymphocyte count | 60.81% (58.35 – 63.27%) | 4-13% |
| NLR | 62.54% (60.15–64.93%) | 4–15% |
| PLR | 52.96% (50.43–55.49%) | 6–12% |
| CRP | 56.78% (54.46–59.10%) | 5–17% |

Values for AUC are presented as percentages with 95% confidence intervals. Higher AUC values indicate better discriminative ability. DCA thresholds denote the range of risk probabilities at which each biomarker provides a net clinical benefit compared with default strategies (treat-all or treat-none). GLR demonstrated the highest discriminative performance for both all-cause and CVD mortality, followed by glucose and NLR, whereas PLR and CRP showed limited predictive value. DCA thresholds were generally wider for all-cause mortality than for CVD mortality, reflecting broader clinical utility across risk ranges.

GLR, glucose-to-lymphocyte ratio; NLR, neutrophil-to-lymphocyte ratio; PLR, platelet-to-lymphocyte ratio; CRP, C-reactive protein; CVD, cardiovascular disease.

**Table S14 Incremental Value of GLR for Mortality Risk Stratification Beyond the Basic Framingham Model.**

| **Variable** | **Continuous NRI(95%CI)** | ***P-*value** | **IDI(95%CI)** | ***P-*value** |
| --- | --- | --- | --- | --- |
| **All cause mortality** |  |  |  |  |
| Basic model | Ref |  | Ref |  |
| Basic model+ GLR | 0.122(0.083-0.159) | 0 | 0.006(0.003 -0.010) | 0 |
| **CVD mortality** |  |  |  |  |
| Basic model | Ref |  | Ref |  |
| Basic model+ GLR | 0.146(0.086-0.207) | 0 | 0.007(0.003-0.012) | 0 |

The basic model: Framingham model (including age, sex, systolic blood pressure, antihypertensive use, total cholesterol, HDL-C, smoking status, and diabetes) was used as the reference model.

Continuous net reclassification improvement (NRI) and integrated discrimination improvement (IDI) are presented with 95% confidence intervals. P-values indicate the statistical significance of changes relative to the basic Framingham model. The addition of GLR led to improvements in risk classification and discrimination for both all-cause and CVD mortality.

GLR, glucose-to-lymphocyte ratio; NRI, net reclassification improvement; IDI, integrated discrimination improvement; CVD, cardiovascular disease.

**Table S15 E-values for the Associations of GLR With All-Cause and CVD Mortality.**

| **Variable** | **E-value for point estimate** | **E-value for lower CI bound** |
| --- | --- | --- |
| **All cause mortality** |  |  |
| GLR(log_2_) continuous | 1.99 | 1.81 |
| GLR(log_2_) tertile | 1.69 | 1.43 |
| **CVD mortality** |  |  |
| GLR(log_2_) continuous | 2.38 | 2.06 |
| GLR(log_2_) tertile | 1.99 | 1.51 |

E-values are presented for the point estimates and for the lower bound of the 95% confidence intervals. Larger E-values indicate that a stronger unmeasured confounder would be required to fully explain away the observed association. The analyzes were based on the fully adjusted Cox regression models.

GLR, glucose-to-lymphocyte ratio; CVD, cardiovascular disease.

**Figure legends**

**Figure S1 Mediation by Systolic Blood Pressure (SBP) of the GLR–mortality link: all-cause and CVD mortality.**

Panel A: Mediation analysis evaluating the indirect effect of systolic blood pressure (SBP) on the association between GLR and all-cause mortality. The total effect of GLR on all-cause mortality was β = 0.0124, with a small but statistically significant indirect effect via SBP (β ≈ 1.0×10^-4^), accounting for 0.95% of the total effect (P = 0.042).

Panel B: Mediation analysis examining SBP as a mediator between GLR and CVD mortality. The total effect of GLR on CVD mortality was β = 0.0028, while the indirect effect through SBP was negligible (β ≈ 0), contributing 0.81% of the total effect (P = 0.042).

**Figure S2 Decision Curve Analysis for the four models.**

Panel A: the net-benefit curves for all-cause mortality; Panel B: the net-benefit curves for CVD mortality.

The figure compares the calibration performance of four progressively adjusted models:

Model 1: no adjusted covariates.

Model 2: adjusted for age, sex, race and ethnicity, educational status, PIR.

Model 3: adjusted for model 2 plus BMI, alcohol consumption, smoke status, hypertension, stroke, CHD, cancer.

Model 4: adjusted for model 3 plus creatinine, BUN, TC, HDL-C, antidiabetic medication use, antihypertensive use, statin use and physical activity.

Across clinically relevant threshold probabilities, Models 3 and 4 yielded consistently higher net benefit compared with Models 1 and 2, with Model 4 providing the greatest overall net benefit. At extreme thresholds, the curves converged toward the "treat-none" strategy, indicating minimal incremental value. These findings suggest that more comprehensively adjusted models improve clinical utility for predicting both all-cause and CVD mortality.

**Figure S3 Calibration plots for the four models.**

Panel A: Calibration curves for all-cause mortality prediction models; Panel B: Calibration curves for cardiovascular disease (CVD) mortality prediction models.

The figure compares the calibration performance of four progressively adjusted models:

Model 1: no adjusted covariates.

Model 2: adjusted for age, sex, race and ethnicity, educational status, PIR.

Model 3: adjusted for model 2 plus BMI, alcohol consumption, smoke status, hypertension, stroke, CHD, cancer.

Model 4: adjusted for model 3 plus creatinine, BUN, TC, HDL-C, antidiabetic medication use, antihypertensive use, statin use and physical activity.

The dashed diagonal line represents perfect calibration. Colored curves depict model-specific calibration trajectories. Curves closer to the diagonal indicate superior agreement between predicted and observed risks. Increased covariate adjustment (Models 3–4) generally improves calibration at the expense of model complexity.
